# A nested-block scalable framework for efficient and equitable urban infrastructure management

**DOI:** 10.1038/s44172-026-00729-x

**Published:** 2026-07-13

**Authors:** Peiyuan Lin, Xiangwei Yu, Tong Liu, Xun Yuan, Chuhao Long, Yang Chen, Haoyi Li, Jun Xiao, Baosong Ma, Linchong Huang, Xian-Xun Yuan

**Affiliations:** 1 School of Civil Engineering, Sun Yat-Sen University, Southern Marine Science and Engineering Guangdong Laboratory, State Key Laboratory of Tunnel Engineering, Zhuhai, China; 2 Department of Civil Engineering, Toronto Metropolitan University, Toronto, ON Canada

**Keywords:** Civil engineering, Computational science

## Abstract

Global cities face mounting challenges in sustaining vast, interdependent infrastructure networks under fiscal and environmental pressures. Conventional optimization methods fail under the dimensional explosion of multi-year maintenance and renewal planning for hundreds of thousands to millions of assets. We present a nested-block optimization framework that decomposes city-wide networks into coordinated sub-blocks, transforming an intractable decision space into a solvable, parallelizable structure while preserving budgetary and temporal constraints. At million-asset scale, the framework achieves rapid convergence and fully feasible plans, far surpassing existing methods in efficiency and scalability. Its architecture unifies diverse assets including roads and pipelines, extends to energy or transit systems, and enables cross-asset, cross-scenario coordination through clustering by district, jurisdiction, or asset class. The framework provides a systematic tool for identifying key evolutionary patterns, evaluating investment pathways, and anticipating system transition conditions, while embedding equity objectives alongside efficiency. These results offer a scientific perspective on urban evolution and practical pathways toward more coordinated, just, and sustainable infrastructure management.

## Abstract (web summary)

Peiyuan Lin and colleagues present a nested-block optimization framework that makes city-wide infrastructure maintenance planning tractable for millions of assets. It rapidly converges where conventional methods fail, explicitly quantifies efficiency–equity trade-offs, and offers a tunable tool for evidence-based infrastructure governance.

## Introduction

Cities worldwide are under mounting pressure to sustain infrastructure networks that are vast, interdependent, and aging^1–4^. Fiscal austerity, climate change, and rising demands for equitable and environmentally responsible services intensify the challenges of maintaining roads, pipelines, and power systems^5–11^. These networks are typically divided among agencies along administrative boundaries, yet in practice they are deeply coupled in both physical structure and functional operation^12–15^. Strategic missteps in this setting may take the form of flawed planning, unbalanced investment, inefficient operations, or fiscal rules that misalign with performance and equity objectives^16–18^. For example, disjointed governance typically manifests when a transportation agency rehabilitates a road, only for an independent water utility to excavate it months later to replace an underlying pipe. Because these physically coupled assets remain administratively and financially siloed, such unsynchronized planning inevitably leads to premature pavement deterioration, wasted capital, and unnecessary service disruptions. Such missteps often cascade into system-wide inefficiencies, rising costs, uneven service, and reduced resiliency^19–21^.

From a formal perspective, city-scale maintenance and renewal planning constitutes a nonlinear integer optimization problem subject to budgetary and scheduling constraints^22–26^. Decision variables multiply across asset inventories, condition states, intervention measures, temporal dependencies, and cross-system interactions, producing a combinatorial explosion of the state space^27–30^. For example, even in a highly simplified scenario with just 100 assets over a 10-year horizon and four annual intervention options per asset, the theoretical number of combinations already reaches ${4}^{{\mathrm{1,000}}}$. This is before accounting for dynamic state transitions, temporal dependencies, budget constraints, and cross-system interactions. Incorporating these real-world complexities will further increase the decision space exponentially. When scaled to millions of assets, this solution space expands explosively to ${4}^{{\mathrm{10,000,000}}}$. With such an astronomical level of complexity that far exceeds human perception, both conventional and advanced algorithms frequently encounter excessive runtimes, nonconvergence, and infeasible solutions^31–33^. The absence of scalable optimization frameworks has made integrated citywide planning nearly unattainable.

Previous research has largely focused on small-scale, single-asset optimization, such as pavement management or pipeline replacement, while giving limited attention to the realities of fragmented governance and system coupling^34–39^. This gap has two consequences. First, there is a lack of tools capable of revealing how multi-asset networks behave under different spatial scales, administrative partitioning, and governance modes. Second, there is no established methodology to quantify the trade-offs between efficiency and equity. As a result, long-term investment strategies are often shaped by narrow perspectives rather than grounded in system-level evidence.

To address these challenges, we develop a genetic algorithm (GA) based nested-block optimization framework (abbreviated as NBGA). This framework organizes urban infrastructure into block-level planning units based on governance structures, such as managerial jurisdictions and administrative boundaries, and coordinates heterogeneous assets through shared budgets and block-level decision units. NBGA scales effectively to millions of assets and reveals how the partitioning granularity of decision-making computational blocks, the system optimization efficiency oriented toward the overall condition of the asset inventory, and the equity of asset condition distribution collectively shape infrastructure governance. It thus provides a scientific basis for the design of more coherent and sustainable models of infrastructure governance.

## Results

### Scalability and feasibility of the nested-block framework

**Fig. 1:**
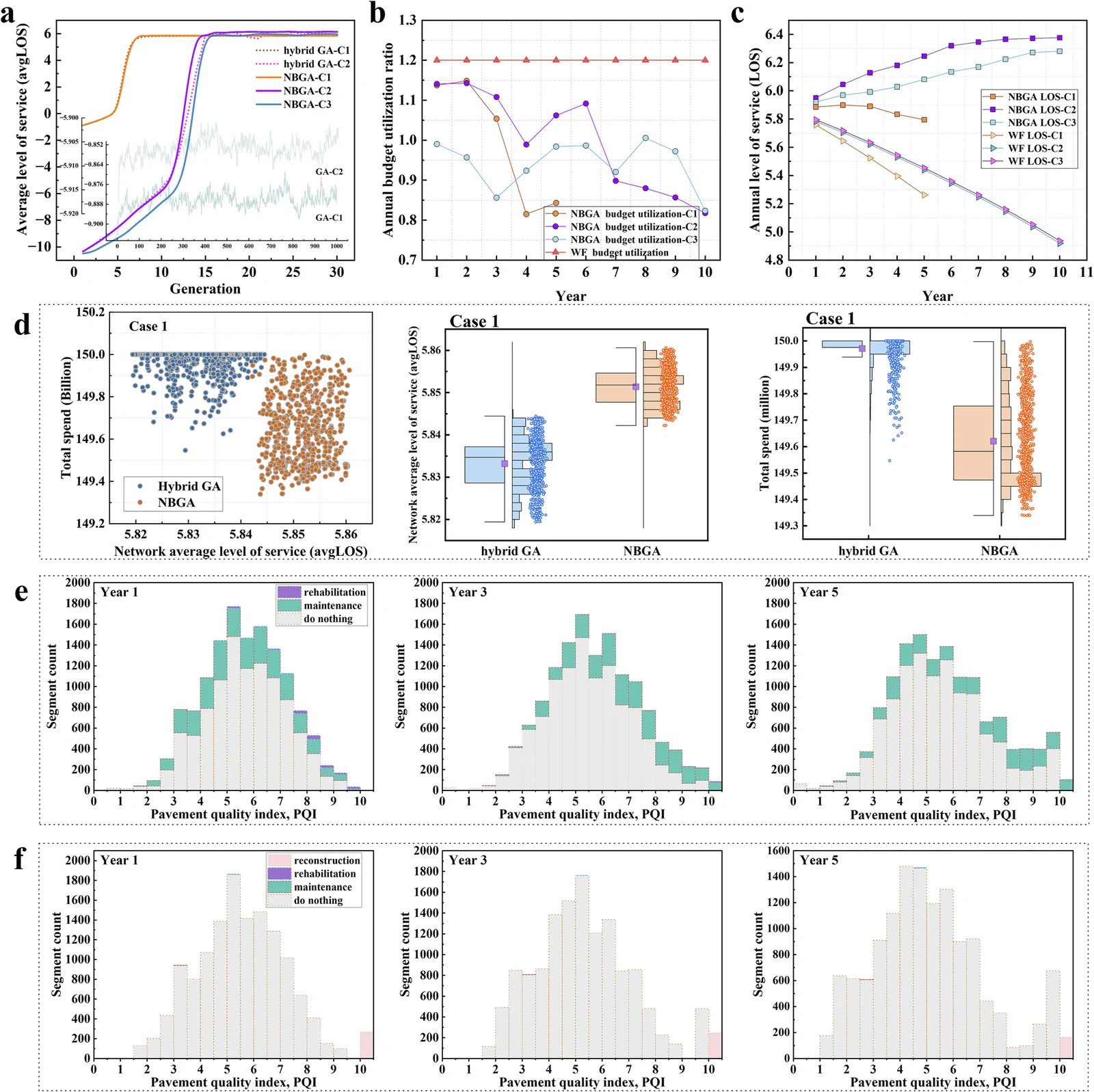
Performance of optimization algorithms and infrastructure condition evolution. **a** Convergence performance of the hybrid GA, NBGA, and standard GA algorithms, measured by the average level of service (avgLOS) per generation. **b** Annual budget utilization ratio over the planning horizon, comparing NBGA across three cases (C1, C2, C3) and the worst-first (WF) baseline. **c** Evolution of the annual level of service (LOS) over the planning horizon for the three case studies under NBGA and WF strategies. **d** Robustness analysis for Case 1 across 100 independent runs. **e** Pavement quality index (PQI) distribution and corresponding optimal decisions produced by NBGA in Years 1, 3, and 5 for Case 1. **f** PQI distribution and corresponding optimal decisions produced by WF in Years 1, 3, and 5 for Case 1.

The mathematical formulation of the NBGA framework is provided in detail in the Methods section later in this article. We systematically evaluated the scalability of the framework through progressively more demanding test cases. In the road asset system of a major North American city (Case 1), featuring an inventory of 13,610 segments (4417 km) and a multi-year decision space of ${4}^{{\mathrm{68,050}}}$ potential solutions. As illustrated by the evolution trajectory of the population’s mean avgLOS (average level of service, the weighted average of the asset conditions over the planning horizon, Eq. 1) in Fig. 1a, the framework converged rapidly and stably, in stark contrast to conventional GAs that oscillated and hybrid approaches that converged far more slowly. This evolutionary path vividly illustrates the algorithm’s capability to swiftly and reliably drive the entire solution set from an initial stochastic infeasible state into a high-quality feasible region. Generational feasibility tracking data (Fig. S5a) further substantiates that the framework achieves high population-wide feasibility upon convergence, and all final solutions preserved in the ‘Hall-of-Fame’ (HOF, comprising ten elite solutions per run) consistently reached a 100% feasibility rate.

Over the entire planning horizon, the framework demonstrates dynamic cross-year resource allocation. Instead of rigidly maxing out annual limits, NBGA flexibly adjusts budget utilization across different years while ensuring adherence to overall budgetary constraints (Fig. 1b), maintaining the asset annual LOS within a steadily improving and controlled range (Fig. 1c). Through global spatiotemporal coordination, NBGA maximally mitigates the natural deterioration of assets and ensures a balanced distribution of pavement health (Fig. 1e and Fig. S6), effectively preventing anomalies or extreme degradation. Valid plans were produced in 6300 s, nearly twice as fast as the hybrid GA (Table S1). In contrast, the conventional worst-first rolling-horizon plan (WF) approach lacked multi-year global optimization, exhausting its annual budget limits (Fig. 1b). This short-sightedness caused severe, continuous LOS declines (Fig. 1c) and trapped the system in a reactive firefighting mode. Operating on a run-to-failure pattern resulted in a persistently high volume of costly reconstruction interventions year after year (Fig. 1f and Fig. S7).

Robustness is a critical prerequisite for translating computational models into practical policy tools. We performed independent parallel experiments using different random seeds for Case 1 (Fig. 1d and Table S1). The empirical evidence demonstrates that, despite varying initial search paths, NBGA consistently yields highly stable solution sets. More importantly, compared to the hybrid GA, NBGA produces solution sets with significantly higher LOS and a lower median total spend. Furthermore, while hyperparameters, such as population size, crossover, and mutation rates typically influence the performance of evolutionary algorithms, our sensitivity analysis indicates that NBGA remains stable and performs consistently across a broad range of parameter settings (Fig. S10 and Table S2). This suggests that in practical applications, decision-makers can rely on standard evolutionary heuristics for parameter selection without the need for exhaustive, costly trial-and-error tuning.

The architectural superiority of NBGA is most vividly demonstrated as the physical asset scale and temporal horizon expand. In a ten-year planning scenario for 100,000 assets (Case 2, decision space of 4^1,000,000^solutions), constructed via tiling of Case 1 data, the algorithm achieved steady-state convergence in just 9 h on a single workstation. Annual LOS and budget allocations remained strictly feasible throughout (Fig. 1a–c and Fig. S8). While this pure tiling approach effectively validates the framework’s computational scalability, it is imperative to verify that such rapid convergence does not merely exploit the high data homogeneity inherent in duplicated datasets. To rigorously address this, we proactively introduced heterogeneity-aware bootstrapping (through perturbed tiling). By applying random perturbations to asset conditions and costs within each block to break data uniformity, and simultaneously implementing non-uniform block partitioning to create structural asymmetry, we stress-tested the framework’s adaptability. The results confirm that NBGA maintains its excellent capability in these highly heterogeneous and asymmetric environments (see Supplementary Text and Figs. S11, 12).

At the million-asset scale, NBGA continued to deliver stable convergence within 20 generations and achieved a steady-state avgLOS of 6.19 (Fig. 1a). The annual expenditure and asset condition across the entire portfolio exhibited stable, healthy normal distributions (Fig. 1b, c and Fig. S8). Even at this large scale, computation remained tractable (291, 400 s, 80 h), whereas competing algorithms diverged or crashed (Table S1). Across all cases, the NBGA solutions were universally feasible across multiple independent runs, and the resulting solutions consistently clustered tightly within the feasible region (Fig. S5). Collectively, these results establish NBGA as a transformative framework that decisively overcomes the curse of dimensionality, extending solvable multi-year, budget-constrained planning from city-scale inventories to previously intractable million-asset systems.

### Structuring blocks governance for integrated asset management

In large metropolitan areas, critical infrastructure systems are often concentrated. As a result, their management extents frequently overlap with administrative jurisdictions, operational zones, or project implementation boundaries. This provides a practical basis for integrated asset management. Building on this, the nested-block framework organizes large-scale asset portfolios into subregions or planning units aligned with governance and implementation needs, allowing each block to function as a project-like decision unit through shared budgets and block-level decision making. Different block-nesting depths can therefore be interpreted as management hierarchies with different levels of coordination and degrees of subdivision (for their real-world operational logic, see Supplementary Materials, Fig. S2). We systematically compared 18 nested strategies (Table S3), assessing their performance based on solution quality, computation time, and budget stability. A standardized evaluation mapping these disparate physical metrics onto a common normalized scale [0, 1] (Eqs. S1–4) revealed significant trade-offs across different nesting depths.

**Fig. 2:**
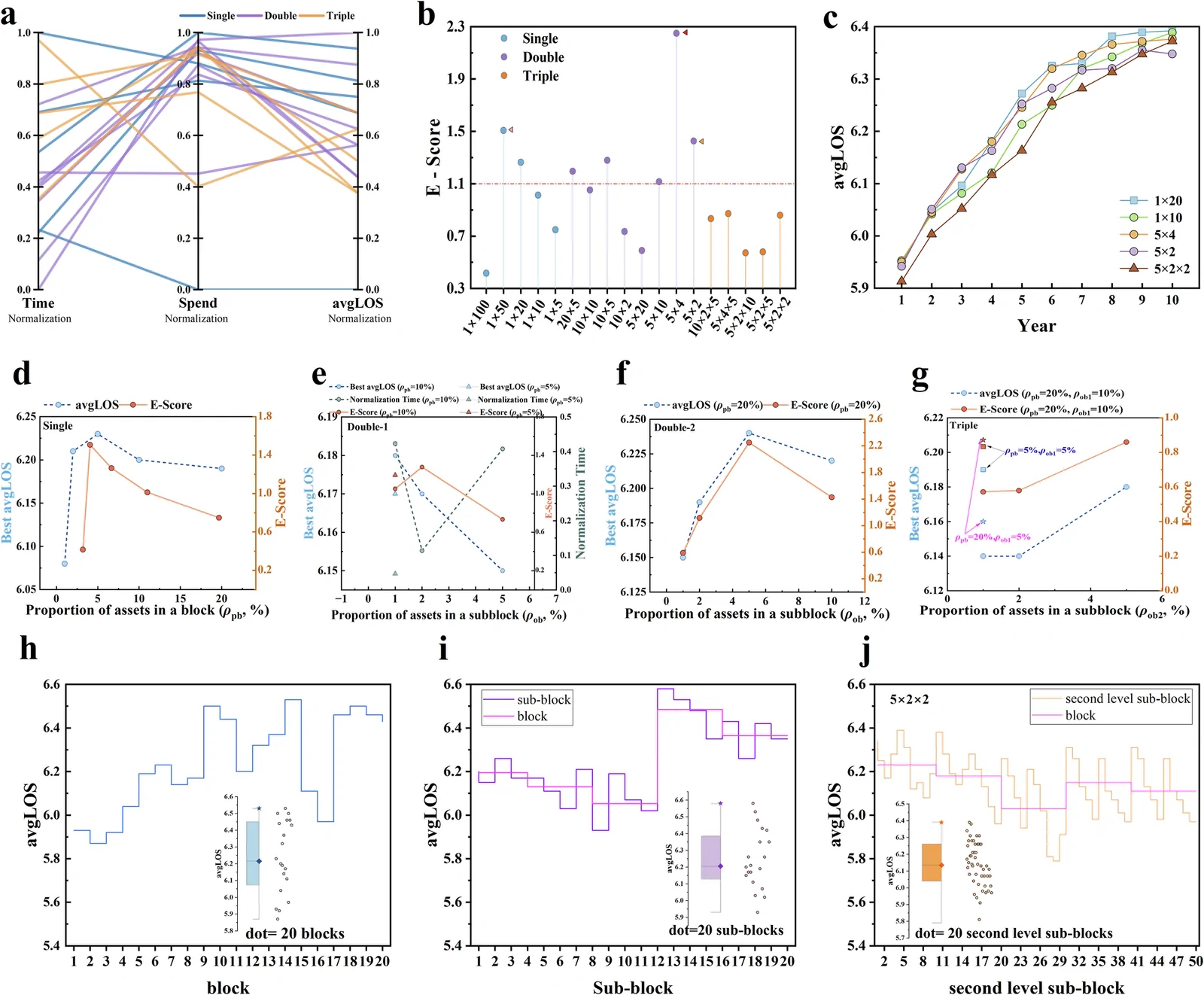
Performance and spatial analysis of hierarchical blocking strategies. **a** Performance comparison (computation time, spend, avgLOS) of Single-, Double-, and Triple-level blocking. **b** E-Score (composite metric) comparison for various blocking configurations. **c** Network-wide avgLOS evolution over 10 years for selected configurations. **d–g** Trade-off analysis for: d Single-level, **e**,**f** Double, and **g** Triple-level strategies. **h** Spatial distribution of avgLOS across the 20 main blocks. The inset boxplot shows the overall variation. **i** Spatial distribution of avgLOS across the 5 × 4 configuration sub-blocks. **j** Spatial distribution of avgLOS across the 5 × 2 × 2 configuration sub-blocks.

Standardized evaluations reveal a clear trade-off between solution quality and computational efficiency. Single-level nesting yields high solution quality (normalized avgLOS ≈1) but markedly slows convergence. Three-level nesting generally induces global inefficiency, slashing normalized avgLOS by over 50%. By contrast, two-level nesting delivers superior balance across the vast majority of scenarios, generating satisfactory solutions in shorter runtimes (Fig. 2a). A unified efficiency score (E-score, which mathematically aggregates normalized solution quality and computational time into a single index; Eq. S4) further confirms the advantage of the two-level design, with the 5 × 4 and 5 × 2 configurations consistently positioned at the forefront of performance rankings (Fig. 2b). Annualized analyses of avgLOS and budget distributions show that two-level schemes maintain high LOS while keeping expenditures stable, whereas weaker designs exhibit larger budget fluctuations and comparatively lower avgLOS (Fig. 2c). Sensitivity tests under the fixed evaluation framework (Fig. 2d–g) highlight the importance of granularity: overly coarse partitions (${\rho }_{ob}$ > 5%) erode computational gains, while overly fine ones (${\rho }_{ob}$ < 1%) over-localize, disrupting cross-asset coordination. For both two- and three-level schemes, performance hinges on interactions between lower-level granularity and upper-level block composition, with optimal ranges concentrated between 1 and 5%.

An assessment of the bottom-tier units generated by each nesting depth, with emphasis on governance alignment and performance patterns, shows that single-level nesting simplifies centralized allocation but masks fine asset-level heterogeneity, producing wide disparities in block-level avgLOS that range from 5.5 to 6.5 (Fig. 2h and Fig. S13). Some blocks inevitably straddle administrative boundaries, complicating coordination. With two levels, differences in avgLOS persist across bottom sub-blocks; yet sub-blocks within the same parent exhibit a similar service level, whereas performance across parents is broadly balanced, with most sub-blocks clustering between 6.2 and 6.5 avgLOS tightly. Higher values concentrate in strategically important, high-weight areas, which is acceptable from an implementation perspective (Fig. 2i and Fig. S14). Three-level nesting introduces finer segmentation, which increases performance variability among the lowest-tier sub-blocks, even though parent-level balance is largely maintained (Fig. 2j). Coordination at neighborhood or node scales remains feasible, but performance diverges more sharply across adjacent small blocks (Fig. S15), yielding inconsistent service delivery between neighboring areas. Aligning many fine partitions with citywide priorities requires more coordination and tighter scheduling rules, increases the risk of local optima, and slightly reduces overall efficiency. Ultimately, these results suggest that perfect alignment with administrative units is a desirable but not absolute objective; limited boundary crossings are an inherent consequence of spatial optimization.

### Fiscal policy controls on compound infrastructure risk dynamics

Urban infrastructure systems continuously balance deterioration and intervention, and investment intensity is the principal factor controlling the long-term trajectory of this evolution. When multiple infrastructure types are incorporated into a unified decision framework, the spatial and temporal allocation of maintenance and renewal investments becomes more complex. The optimization objective correspondingly shifts from improving service levels within single-asset systems to managing compound risk (the network-wide aggregation of individual asset risks, defined as the product of Probability of Failure and event loss) and minimizing cumulative expected loss across co-managed asset systems (Eqs. 7–10). Understanding the fiscal rules that govern this compound risk is essential for effective investment planning. We conducted tests on these dynamics using three integrated asset networks totaling 400,000 assets but with varying compositions: (100k roads, 300k pipes), (200k roads, 200k pipes), and (300k roads, 100k pipes).

**Fig. 3:**
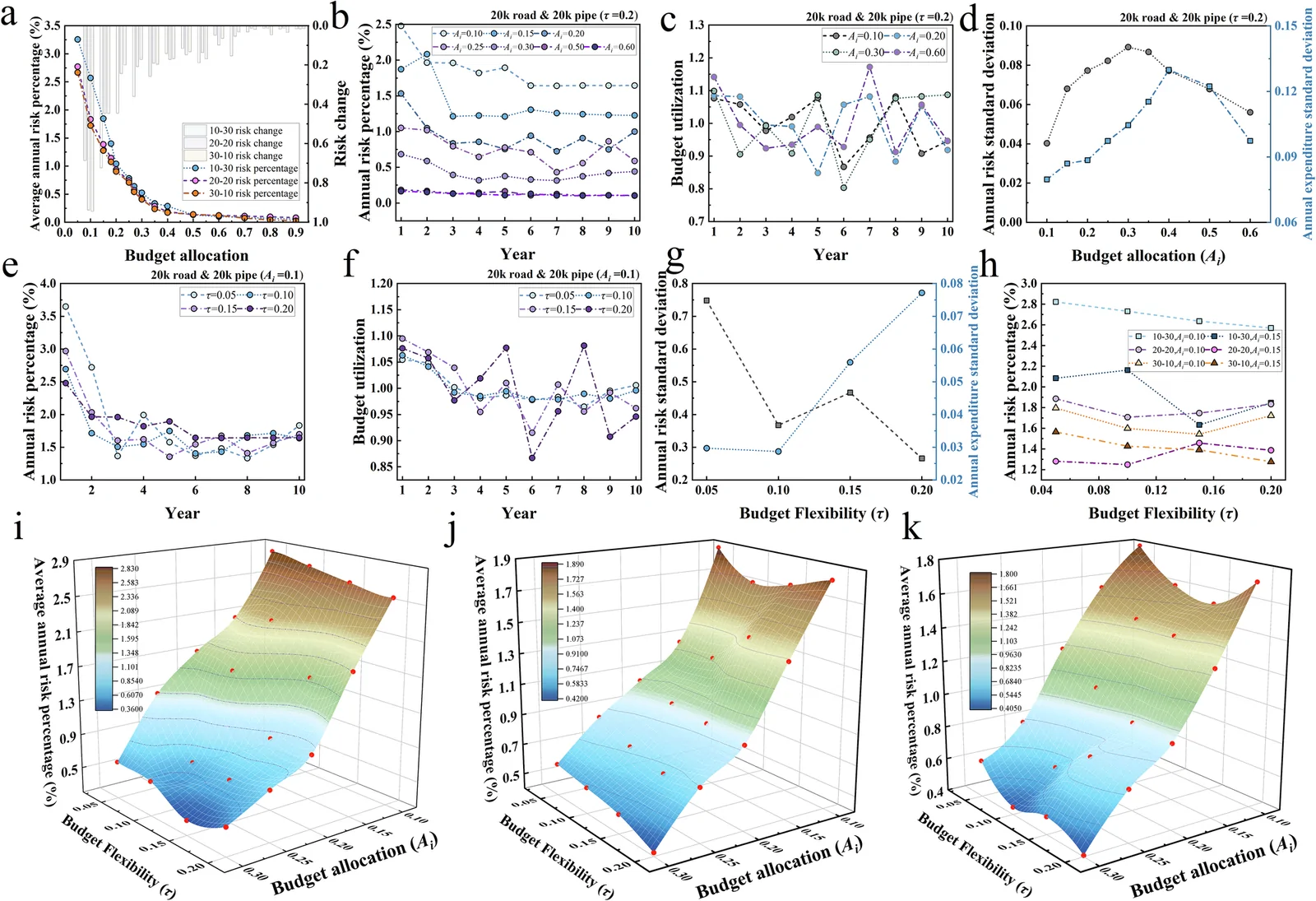
Budget allocation (*A*_*i*_) and flexibility (*τ*) parameters drive network risk and stability. **a** Average annual risk percentage vs. budget allocation for three different asset portfolios (100k/300k, 200k/200k, and 300k/100k road/pipe). **b** Annual risk change over 10 years for different ${A}_{i}$ values. **c** Budget utilization over 10 years for different ${A}_{i}$ values. **d** Risk and expenditure standard deviation as a function of ${A}_{i}$. **e** Annual risk percentage over 10 years for different τ values. **f** Budget utilization over 10 years for different τ values. **g** Risk and expenditure standard deviation as a function of τ. **h** Average annual risk percentage vs. budget flexibility for the three asset portfolios. **i–k** 3D response surfaces of average annual risk as a function of ${A}_{i}$ and τ for the three portfolios: **i** (100k road & 300k pipe), **j** (200k road & 200k pipe), and **k** (300k road & 100k pipe).

Budget allocation (${A}_{i}$), the proportion of total replacement cost invested annually, is the principal determinant of network risk. Analyses of the three portfolios reveal that, for the established networks with ongoing deterioration, allocations at low levels (${A}_{i}\approx 0.1$) tend to be insufficient to offset systemic degradation, which in this case results in network risk as high as 3% of the total annual asset failure loss. As ${A}_{i}$ increases, the system exhibits diminishing marginal returns; for ${A}_{i} > 0.5$, high-priority interventions become saturated, and risk reduction decelerates sharply (risk change <0.05, Fig. 3a). This pursuit of lower risk, however, can introduce significant temporal volatility. The temporal dynamics reveal substantial year-to-year fluctuations in both annual risk (Fig. 3b) and budget utilization (Fig. 3c), indicating underlying instability. This macro-level volatility, which peaks at 10% (Fig. 3d), does not exhibit a monotonic relationship with ${A}_{i}$ but rather a non-linear trend, first increasing and then decreasing. This indicates that volatility is a compound result, controlled not only by allocation but also strongly constrained by other budgetary measures.

We next examined the impact of budget flexibility (τ). To isolate this effect, we kept allocation constant (e.g., at ${A}_{i}$ = 0.1). The temporal dynamics again reveal significant annual volatility in risk and budget utilization. Overly strict limits ($\tau \approx 0.05$) create unstable, oscillating risk profiles (Fig. 3e, f), as the system struggles to shift funds across years to finance urgent, high-cost interventions. Conversely, loose limits (τ=0.20) induce high expenditure volatility but, critically, succeed in maintaining risk stability (Fig. 3g). Counterintuitively, Fig. 3h reveals that network performance is not monotonic with flexibility; an optimal tolerance corridor (τ between 0.05 and 0.15) yields balanced outcomes. This suggests a strong coupling with investment intensity (allocation, ${A}_{i}$), where higher allocations may require different flexibility levels to be effective, necessitating a joint analysis of their coupled relationship.

The 3D response surfaces (Fig. 3i–k) synthesize these dynamics, revealing the coupled effects of ${A}_{i}$ and τ. The topology of the response surface, visibly contoured by the non-linear interaction of allocation and flexibility, reveals a clear ‘valley’ of optimal performance (lowest risk), confirming that neither parameter can be optimized in isolation. This demonstrates that a universal investment threshold is unrealistic and that optimal fiscal policies are portfolio-specific. The framework thus moves beyond simple thresholds to provide a computable method for quantifying these complex trade-offs. It enables managers to simulate and identify robust fiscal policies (i.e., ${A}_{i}-\tau$ combinations) that achieve effective compound risk control and long-term fiscal sustainability.

### Quantifying and mitigating systemic inequity in asset management

**Fig. 4:**
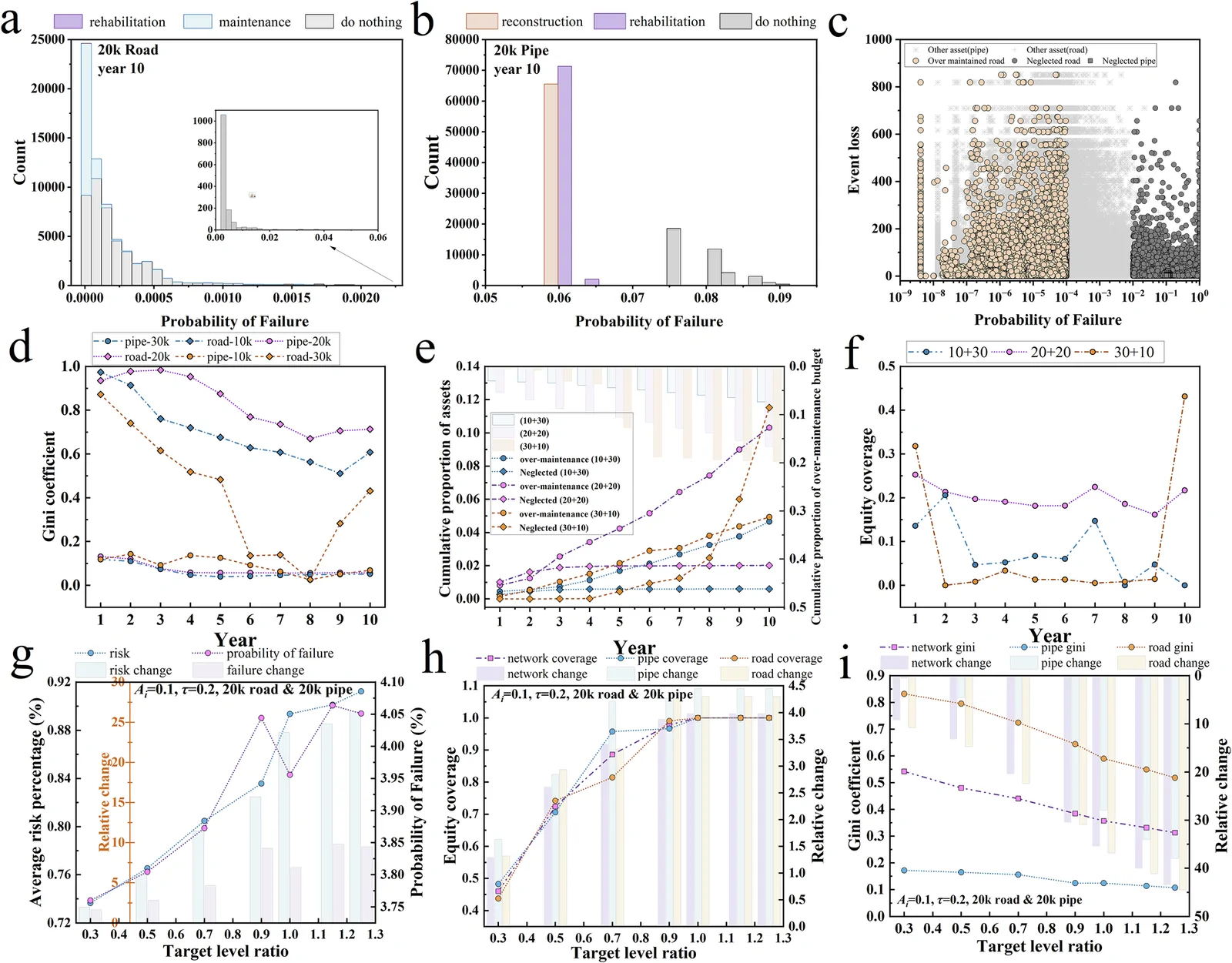
Diagnosing risk inequity and the impact of target-level control. **a** Failure probability distribution for roads. **b** Failure probability distribution for pipes. **c** Event loss vs. failure probability, identifying neglected and over-maintained assets. **d** Gini coefficient (inequity) evolution over 10 years for different portfolios. **e** Cumulative proportion of neglected assets (lines) and their over-maintenance budget share (bars). **f** Equity coverage evolution over 10 years for different portfolios. **g** Average network risk vs. target level ratio. **h** Failure probability and equity coverage vs. target level ratio. **i** Gini coefficient and its relative change vs. target level ratio.

Conventional maintenance optimization, while maximizing network-wide efficiency, often creates systemic inequity. Our simulations reveal that efficiency-driven strategies produce a ‘long tail’ of high-risk assets within the road and pipe network (Fig. 4a, b, and Fig. S16). This leads to a stark performance bifurcation across the asset portfolio (Fig. 4c). A subset of assets becomes ‘neglected’, characterized by high failure probability and high event loss. Simultaneously, another subset is ‘over-maintained’, receiving resources despite low failure probability. Perversely, this over-maintenance extends even to assets with both low event loss and low failure probability, which is anomalous. This finding highlights a core governance conflict: the pursuit of global efficiency not only systematically sidelines vulnerable network components but also generates perverse and untrustworthy allocation outcomes.

This inequity is persistent and self-reinforcing. Over the ten-year planning horizon, the Gini coefficient (a measure of risk inequity quantified by the distributional disparity of failure risks across the asset population, Eq. S6, with values above 0.4 often considered a threshold for high inequality in policy contexts^40^) is highly volatile for road-heavy portfolios, fluctuating widely between 0.2 and 0.9 (Fig. 4d). This instability, which includes sharp temporary drops, is quantified by a large inter-annual volatility (annual deviation σ>0.1, Fig. S17). Concurrently, the cumulative proportion of over-maintained assets steadily increases, reaching a maximum of 10% of the total portfolio by Year 10, while the budget share allocated to this over-maintenance also rises to approximately 20% (Fig. 4e). Furthermore, ‘equity coverage’ (the proportion of neglected assets that receive intervention, Eq. S7) remains not only low, frequently dropping below 10% (Fig. 4f), but is also exceptionally erratic (Fig. S17). These quantify a systemic failure: the efficiency-focused model consistently fails to self-correct its inequitable outcomes.

The framework controls this inequity by embedding an equity-based control, the ‘target level ratio’ (TLR), which scales a minimum tolerable baseline (0.01 probability of failure for roads, 0.1 for pipes). The framework precisely quantifies the resulting efficiency-equity trade-off. Simulations show that a TLR of 0.7 elevates equity coverage to 80% (Fig. 4h) and reduces the Gini coefficient to 0.5 (Fig. 4i). Increasing the TLR to 1.15 reduces inequity by 67% (Gini from 0.9 to 0.3), but this gain is coupled with a modest, quantifiable increase in network risk from 0.75 to 0.90% (Fig. 4g and Fig. S18). This transforms the framework from a simple optimization model into a powerful policy simulator: policymakers can now estimate the expected level of equity (Gini coefficient) for a specific target setting (TLR) and its precise, quantifiable cost (average risk), which is invaluable for evidence-based governance.

## Discussion

The nested-block optimization framework presented here transcends a purely computational contribution: it creates a practical, testable interface between governance architecture and asset-level decision-making^16,18,41^. By recasting a million-asset, multi-year planning problem as a hierarchy of coordinated subproblems, the framework removes the principal computational barrier of combinatorial explosion^31–33^ while retaining the policy constraints and fidelity required for operational deployment. This methodological advance therefore converts previously intractable scenarios into tractable policy experiments, enabling planners to evaluate not only what can be achieved with a given budget, but how organizational design and fiscal rules shape achievable outcomes.

The results demonstrate that spatial governance is an active design variable for optimization rather than a fixed background condition^42,43^. The empirical finding that two-level nesting delivers the most favorable trade-off between solution quality and computational cost provides a concrete, data-driven prescription for harmonizing administrative realities with algorithmic efficiency. This hybrid design preserves sufficient spatial aggregation to exploit economies of scale while maintaining enough local resolution to capture cross-asset heterogeneity and neighborhood-scale priorities. The result challenges polar views that favor either absolute centralization or extreme hyper-localization and instead suggests that effective coordination in large asset systems may depend on planning units that remain both governance-aligned and operationally meaningful^44,45^.

The framework also functions as a fiscal policy laboratory. The exploration of allocation and temporal flexibility parameters shows that risk and volatility emerge from nonlinear, coupled interactions rather than from single parameters in isolation^46^. The response-surface perspective reframes fiscal design as a multi-dimensional search for robust corridors of performance, specifically policy combinations that simultaneously manage average risk, inter-annual volatility, and equity exposure, rather than as single, universal thresholds. In practice, this implies that budget rules and tolerance bands should be co-designed with portfolio composition and strategic priorities^47^.

Most notably, the analyses make the efficiency-equity trade-off explicit and operational^48,49^. Efficiency-oriented optimization reliably concentrates interventions where marginal benefit is highest, but this produces a persistent long tail of neglected, high-risk assets and a distinct class of over-maintained assets. By embedding a transparent target-level control (TLR), the framework converts equity from an aspirational goal into a tunable policy instrument: decision-makers can quantify the equity gains (e.g., reductions in portfolio Gini) and the corresponding, precisely measured efficiency costs (changes in average risk), and thereby make defensible, evidence-based trade-offs^50^. This operationalization facilitates accountability and makes distributive consequences visible before implementation.

The framework is readily extensible. Its modular architecture supports inclusion of additional infrastructural modalities (e.g., power, transit)^51^ and finer socio-spatial metrics^52^, enabling analyses of cascading failure risk^53,54^, multi-sector dependencies, and human exposure metrics. Extending the framework in these directions will enable integrated scenario analyses that combine physical fragility, service disruption impacts, and socio-demographic vulnerability, thereby connecting engineering choices to social outcomes. Beyond expanding this functional scope, deepening the optimization’s physical fidelity and operational granularity constitutes a critical next step. First, while the current architecture leverages spatial bundling for governance coordination, explicitly incorporating physical coupling constraints, such as resurfacing-cut penalties and precise joint packaging logistics, warrants dedicated investigation. Second, because the current framework primarily establishes spatial coordination and budget-level feasibility at the strategic planning scale, integrating complex tactical delivery constraints (e.g., localized crew capacities and seasonal construction windows) will be essential to transition the model toward full operational project scheduling. Such methodological deepening, alongside broader empirical validation across independent infrastructure networks from different regions, will be essential for substantiating the universal applicability of advanced asset management algorithms across diverse geographic, institutional, and socio-technical contexts.

In sum, the nested-block framework moves the field from reactive maintenance and small-scale optimization to prescriptive, system-level governance tools. It provides both the computational tractability needed to plan at an urban scale and the policy levers desired to align investments with public values, including efficiency, fiscal sustainability, and equity. Ultimately, the framework paves a practical pathway for cities to govern complex, interdependent infrastructure systems more coherently and justly.

## Methods

### Mathematical formulation

Consider a spatially distributed asset inventory containing n assets over a planning horizon of h years. Asset i in year t has condition ${\mu }_{{it}}$ or failure probability ${P}_{{it}}$ and receives a maintenance, rehabilitation, or reconstruction action (MRR) ${j}_{{it}}\in \{{\mathrm{0,1}},\ldots ,m\}$, where ${j}_{{it}}=0$ denotes no action.

The performance objective depends on the analytical perspective. For service-oriented planning, the goal is to maximize the average network level of service (avgLOS),

1 $$\max {{\rm{avgLOS}}}=\frac{1}{h}{\sum }_{t=1}^{h}{\sum }_{i=1}^{n}{w}_{i}{\mu }_{{it}}$$

where ${w}_{i}$ represents the weight of asset i (e.g., length or importance). For risk-oriented integration, the objective is to minimize the expected loss,

2 $$\min {{\rm{Risk}}}=\frac{1}{h}{\sum }_{t=1}^{h}{\sum }_{i=1}^{n}{w}_{i}{P}_{{it}}{L}_{{it}}$$

where ${L}_{{it}}$ represents the direct and indirect consequences of failure, ${P}_{{it}}$ represents the probability of failure.

Budgetary feasibility and service delivery are imposed through three constraints. The annual budget must lie within tolerance ${\tau }_{t}$ of the designated budget ${B}_{t}$,

3 $$(1-{\tau }_{t}){B}_{t}\le {\sum }_{i=1}^{n}{c}_{{it}}\le (1+{\tau }_{t}){B}_{t}$$

where ${c}_{{it}}$ is the cost of the MRR action ${j}_{{it}}$; and the total cost must not exceed the horizon budget.

4 $${\sum }_{t=1}^{h}{\sum }_{i=1}^{n}{c}_{{it}}\le {B}_{{total}}$$

and exclusivity requires that each asset takes one and only one action per year. The resulting problem is a constrained nonlinear integer program because the condition evolution and the objective are nonlinear, while the decision variables are discrete.

It is critical to clarify the operational definition of this tolerance band. The annual budget tolerance (Eq. 3) is neither an algorithmic concession nor a heuristic relaxation intended to accommodate infeasible solutions. Rather, it is a rigidly defined fiscal policy parameter that mathematically encodes the necessary flexibility of real-world multi-year budget windows. Within the NBGA framework, the overall horizon budget serves as a strict upper bound. Consequently, as long as the formulated plans fall exactly within these designated annual tolerance windows and do not exceed the total horizon envelope by mathematical definition, they are strictly compliant with the hard constraints.

### Deterioration–maintenance–failure processes

For single-asset systems, or those with homogeneous performance metrics, the objective is to maximize the network-wide average level of service (avgLOS), objective formulation in Eq. 1. For complex, multi-asset networks, the framework employs a risk-oriented integration, where the objective is to minimize the expected loss, objective formulation in Eq. 2.

It is emphasized that the primary focus of this study is the optimization framework. The specific deterioration models and parameters presented below are adopted to demonstrate the framework’s capabilities under representative planning conditions. While the framework is model-agnostic in principle, the quantitative optimization behavior and resulting solutions may vary with the chosen deterioration models, parameter settings, and constraint regimes.

#### Deterministic condition model

For the single-asset (road network) scalability tests, we employ a deterministic condition model based on the Pavement Quality Index (PQI), ranging from 0 (failed) to 10 (new). The network asset condition evolves via a two-stage update that accounts for natural deterioration and the instantaneous effects of interventions, and is governed by a set of parameters^55,56^. The first stage describes natural deterioration as a gradual reduction in condition,

5 $${\mu }_{{it}}=\max ({\mu }_{i\left(t-1\right)}-S\times k\times {({\mu }_{i\left(t-1\right)})}^{k-1},\varepsilon )$$

in which S = a scale parameter; k = a rate parameter; and ε = a near zero threshold to prevent the asset condition from becoming negative or zero. For the purposes of this study, the parameters are set as follows: *S* = 10, *k* = 0.1, and ε = 1×10^−9^. The second stage represents intervention-induced improvement,

6 $${\mu }_{{it}}=\min ({\mu }_{i\left(t-1\right)}+\zeta ,{\mu }_{{it}({{\rm{m}}}{ax})})$$

where ζ represents the instantaneous improvement in ${\mu }_{{it}}$ after implementing the MRR action, and ${\mu }_{{it}({{\rm{m}}}{ax})}$ is the maximum possible value a segment can achieve, which is set to 10 in this study. In this study ζ= 0.8, 3, and 10 for preservation, rehabilitation, and reconstruction, respectively. This two-step formulation captures the nonlinear interplay between deterioration and renewal and preserves the annual continuity of the condition state.

#### Stochastic risk model

To conduct the multi-asset (road and pipe) compound risk and equity analyses, we use a continuous-time stochastic process. This model is applied to both road segments and pipe segments.

To represent the stochastic nature of structural degradation and failure, deterioration and risk evolution are further characterized by continuous-time stochastic processes. For the progressive and monotonic accumulation of damage to a structure condition (e.g., material fatigue, pavement wear, or pipe corrosion), the core assumption is that the cumulative damage over a one-year period is a random variable X, which follows a Gamma distribution $X \sim {Gamma}(\alpha ,\theta )$^57^, where α is the shape parameter and θ is the scale parameter.

A structural ‘failure’ is defined as the event where its performance condition metric falls below a predefined critical threshold ${C}_{{fail}}$ by the end of the year. The annual probability of this failure is calculated based on the condition (${\mu }_{i,t+1}$) at the start of the year after a maintenance action ${j}_{{it}}$. For repair-type actions, the condition improves by an amount ${\delta }_{j}$, resulting in a state of ${\mu }_{i,t+1}={\mu }_{{it}}+{\delta }_{j}$; for renewal-type actions, the condition is reset directly to its maximum value ${\mu }_{{it}({{\rm{m}}}{ax})}$. Based on this post-maintenance condition, the damage threshold that must be exceeded within the year to cause a failure is $d={\mu }_{i,t+1}-{C}_{{fail}}$. Therefore, the structure annual failure probability (PoF) ${P}_{f}(i,t)$ is defined as the probability that the annual damage increment X exceeds this threshold d, calculated by the equation:

7 $${P}_{f}(i,t)=P\left(X\ge d\right)={\int }_{d}^{\infty }\frac{{x}^{\alpha -1}{e}^{-x/\theta }}{{\int }_{0}^{\infty }{t}^{\alpha -1}{e}^{-t}{dt}{\theta }^{\alpha }}{dx}$$

For discrete, sudden failure events (e.g., equipment breakdown or pipe bursts), the model employs a Non-Homogeneous Poisson Process (NHPP). The core of this model is that the failure intensity varies dynamically with the asset service age t. For a specific asset i, its cumulative intensity function is defined as:

8 $${\Lambda }_{i}\left(t\right)={M}_{i}{t}^{\beta }$$

where β is the shape parameter that dictates the trend of the failure rate over time, and ${M}_{i}$ is a scale factor unique to the asset, reflecting its physical attributes. The expected number of failures over a one-year period (from age ${t}_{y}$ to ${t}_{y}+1$) is the increment:

9 $$\Delta {\Lambda }_{i}\left({t}_{y}\right)={\Lambda }_{i}\left({t}_{y}+1\right)-{\Lambda }_{i}\left({t}_{y}\right)={M}_{i}({\left({t}_{y}+1\right)}^{\beta }-{t}_{y}^{\beta })$$

Consequently, the probability of at least one failure occurring within that year is:

10 $${P}_{f}(i,{t}_{y})=1-{e}^{-\Delta {\Lambda }_{i}({t}_{y})}$$

Maintenance actions ${j}_{{it}}$, directly influence the calculation of this annual failure probability. For a minimal repair, the original expected number of failures is multiplied by a repair factor ${\rho }_{{patch}}$ (ranging from 0 to 1). For an imperfect repair, the current age ${t}_{y}$ is first reduced to an effective age ${t}_{y}^{,}={\kappa }_{{lining}}\cdot {t}_{y}$ by a rejuvenation factor ${\kappa }_{{lining}}$ (ranging from 0 to 1), and the expected number of failures is calculated based on this younger effective age before being multiplied by another repair factor ${\rho }_{{lin}}$ (ranging from 0 to 1). For a full renewal, the asset is treated as ‘as-good-as-new’, and its expected number of failures for the year is calculated based on an age of zero. This modeling choice is adopted in the present case study and affects the resulting risk estimates and optimization outcomes under this formulation, but does not alter the framework's general nested-block search architecture. Therefore, this study uses fixed factors throughout: ${\rho }_{{patch}}=0.3$, ${\kappa }_{{lining}}=0.6$, and ${\rho }_{{lin}}=0.6$.

### Nested block optimization framework

We represent each candidate solution as the full action matrix {${j}_{{it}}$} over the planning horizon h. Global exploration begins with annual plan swapping: a year ${t}^{* }$ is sampled and the entire column ${\left\{{j}_{{{it}}^{* }}\right\}}_{i=1}^{m}$ is exchanged between two parents to create two offspring, which preserves annual budget structure and within-year logic (Fig. S1a).

Within a selected year, the asset set is blocked and nested into governance-aligned or region-aligned units. Assets are first partitioned into blocks ${b}_{f}$ and based on asset attributes or jurisdiction, are recursively split into sub blocks ${b}_{{fs}}$ until the end level permits coordinated management of assets within each block (Fig. S1b). Budgets for the year are allocated to sub-blocks in proportion to weights ${\sum }_{i\in {b}_{{fs}}}{w}_{i}$ so that sub-block allocations sum to the annual budget ${B}_{t}$. Searching on this hierarchy combines block crossover and block LP mutation. Block crossover transfers compact groups of decisions between sub-blocks to propagate effective spatial motifs without dismantling feasible structure (Fig. S1c). Sub-blocks are scored by an improvement potential value (${{{\rm{IPV}}}}_{{{\rm{s}}}}$). Exchanges focus on mid-rank sub-blocks by ${{{\rm{IPV}}}}_{{{\rm{s}}}}$ to maintain diversity and to avoid premature convergence.

Refinement is carried out by block LP mutation at the end level (Fig. S1d). For sub-block ${b}_{{fs}}$ in year t we maximize ${{LOS}}_{t,s}={\sum }_{i\in {b}_{{fs}}}{w}_{i}{\mu }_{{it}}$ or minimize ${{Risk}}_{t,s}={\sum}_{i\in {b}_{{fs}}}{w}_{i}{P}_{{it}}{L}_{{it}}$, subject to the local budget bounds. Linear programs are solved only for the captured set of the top $K={\sum }_{s}{P}_{s}$ sub-blocks, which are selected according to their ${{{\rm{IPV}}}}_{s}$ and the size-weighted probability ${P}_{s}$. If some sub-blocks become feasible through crossover or mutation, their allocations are fixed, and the remaining annual budget is redistributed proportionally before the next refinement.

### Blocking and nesting operations

The genetic operations for NBGA are as follows (c.f. Fig. S1).

(i) APS Crossover: this operation is the same as the one proposed by Fard and Yuan^33^ for the enhanced hybrid GA. A year is randomly selected, and the annual plan for that year in the chromosomes of the two parents is swapped to generate two offspring.
(ii) Blocking and nesting: the core operations, such as blocking, nesting, crossover, and mutation, are applied to an infeasible planning year. If all annual plans are already feasible, then a year is randomly selected for subsequent operations. If there are multiple infeasible annual plans, then the first infeasible plan in chronological order is selected for subsequent operations.

The blocking and nesting process, decomposes a large maintenance operation vector into a multi-level, block-based hierarchical structure to accelerate the solution process. It is important to note that this process also incorporates an MRR mutation mechanism. This mutation is analogous to the mutation operator in GA, but is more constrained to allow for a controlled exploration of the solution space. Specifically, for a given block ${b}_{f}$ containing ${n}_{f}$ assets, a number of assets (mutation rate×${n}_{f}$) are randomly selected. Their assigned MRR actions are then forcibly changed to other arbitrary actions.

The interaction between the MRR mutation and the next block crossover operation is determined by the nesting depth, following a recursive logic:

1. level 1 (One Level of Nesting): if a block ${b}_{f}$ is not partitioned into sub-blocks, it first undergoes MRR mutation. The mutated block then proceeds to the block crossover stage and other subsequent operations.
2. level 2 (Double Level of Nesting): if a block ${b}_{f}$ is partitioned into sub-blocks (${b}_{{fs}}$), the parent block ${b}_{f}$ first undergoes MRR mutation. Afterward, block crossover and other subsequent operations are performed among its sub-blocks ${b}_{{fs}}$.
3. level 3 and deeper (Recursive Nesting): this pattern continues recursively. For instance, if a sub-block ${b}_{{fs}}$ is further partitioned into second-level sub-blocks ${b}_{{fsl}}$, the process is as follows:

The parent block ${b}_{f}$ undergoes MRR mutation;

Block crossover is performed on its sub-blocks ${b}_{{fs}}$;

Each sub-block ${b}_{{fs}}$ then undergoes its own MRR mutation;

Finally, block crossover is performed on the second-level sub-blocks ${b}_{{fsl}}$.

This hierarchical application of mutation and crossover is consistently applied to any further levels of nesting (e.g., fourth, fifth), ensuring a structured and scalable search strategy.

(iii) Block crossover: this operation is designed to operate between sub-blocks and differs significantly from GA or APS crossover operators. Its function is more akin to migration in an evolutionary ‘Island Model’^58^, where distinct populations (here, sub-blocks) evolve in parallel and periodically exchange individuals to enhance the global search. This parallel evolution is realized through independent MRR and Block-LP mutations within each block post-nesting.
  In the implementation of the double level of nesting, the following procedure is applied. First, from each subblock ${b}_{{fs}}$, groups with mid-range rankings based on their Improvement Potential Value (${{{\rm{IPV}}}}_{s}$) are selected. The assets within these selected subblocks are then designated for exchange. These subblocks are randomly shuffled and conceptually arranged in a ring, allowing the last sub-block to be paired with the first. Finally, these paired subblocks execute MRR actions to swap their designated assets, thereby facilitating the transfer of solution features across the population. The IPV is calculated using the following equation:
  11 $${{{\rm{IPV}}}}_{s}=-{\sum }_{i=1}^{{n}_{{fs}}}{w}_{i}({\mu }_{{it}(\max )}-{\mu }_{{it}})$$
(iv) Block-LP mutation: implemented at the end level of subblocks. The linear programming (LP) technique is applied to each subblock, subject to its corresponding budget. The specific equation depends on the system optimization objective:

1. for the single-asset systems, or those with homogeneous performance metrics, the objective is to maximize the average level of service (avgLOS):
  12 $$\max {{\rm{LO}}}{{{\rm{S}}}}_{t,s}={\sum }_{i=1}^{{n}_{{fs}}}{w}_{i}{\mu }_{{it},s}$$
2. for complex, multi-asset networks, the framework employs a risk-oriented integration, where the objective is to minimize the expected cumulative loss:

13 $$\min {{{\rm{Risk}}}}_{t,s}={\sum }_{i=1}^{{n}_{{fs}}}{{w}_{i}P}_{{it}}{L}_{{it}}$$

14 $${{\rm{subject\; to}}}(1-{\tau }_{t}){B}_{t,s}\le {\sum }_{i=1}^{{n}_{{fs}}}{c}_{{it},s}\le (1+{\tau }_{t}){B}_{t,s}$$

where the subscript s in Eqs. 12–14 denotes that the mutation operation is carried out on an end-level subblock ${b}_{{fs}}$. Parameter ${n}_{{fs}}$ is the number of assets included in the subblock ${b}_{{fs}}$; ${\mu }_{{it},s}$ and ${c}_{{it},s}$ are the condition and MRR cost of an asset ${x}_{{it}}$ in ${b}_{{fs}}$, respectively; ${LO}{S}_{t,s}$ is the sum of the LOS of the entire subblock ${b}_{{fs}}$ in year t; ${B}_{t,s}$ is the budget allocated to the subblock ${b}_{{fs}}$, which is determined by:

15 $${B}_{t,s}=\frac{{\sum }_{i=1}^{{n}_{{fs}}}{w}_{i}}{{\sum }_{i=1}^{n}{w}_{i}}{B}_{t}$$

where n is the total number of assets.

The block-LP mutation is a targeted refinement operator applied exclusively to subblocks that are identified as infeasible. If a subblock already satisfies its constraints, potentially due to preceding mutation or crossover operations, this LP-based refinement is bypassed to conserve computational resources. For the remaining infeasible subblocks, a ‘capture’ phase is initiated to select a promising subset ${b}_{{fs},{cap}}$ for optimization.

This selection is a two-stage procedure. First, a target number of subblocks to optimize *K* is dynamically calculated. In practice, the blocking operation can partition assets by category, characteristics, geographic location, or project affiliation, typically resulting in subblocks of heterogeneous sizes. Smaller subblocks correspond to problem instances with fewer variables and a narrower scope, which are generally easier to optimize and more likely to yield rapid improvement, whereas larger subblocks are more complex and computationally demanding. To reflect this difference, each subblock ${b}_{{fs}}$ is assigned a base selection probability ${P}_{s}$ inversely related to its size ${n}_{{fs}}$.

16 $${P}_{s}={\rho }_{\min }+({\rho }_{\max }-{\rho }_{\min })\frac{1}{{\mathrm{ln}}({n}_{{fs}})}$$

where ${\rho }_{\min }$ and ${\rho }_{\max }$ are the lower and upper bounds, in this research ${\rho }_{\min }=0.4$ and ${\rho }_{\max }=0.9$. The *K* is then determined by taking the ceiling of the sum of probabilities for these *p* subblocks.

17 $$K=\left[{\sum }_{s=1}^{p}{P}_{s}\right]$$

When many small subblocks exist, their larger ${P}_{s}$ values cause the sum and thus *K* to increase. In the subsequent worst-first (WF) selection, this allows more subblocks that are both small in size and high in improvement potential to be chosen for execution, thereby preventing promising small subblocks from being excluded while also maintaining computational efficiency. In contrast, if all subblocks are of equal size, ${P}_{s}$ would be identical for every block, and *K* could be directly obtained by multiplying this uniform probability by the total number of subblocks.

Second, once *K* is established, the specific subblocks are chosen based on their IPVs. All infeasible subblocks are ranked by their IPVs, and the top *K* candidates, which are those with the highest potential for improvement, are selected, analogous to a worst-first (WF) strategy.

Consequently, the budget for the LP Mutation procedure must be adjusted to account for the resources already allocated to the feasible subblocks ${B}_{t,f}$. The new budget for each selected subblock is proportionally distributed from the remaining global budget:

18 $${B}_{t,s}^{{\prime}}=\frac{{\sum }_{i=1}^{{n}_{{fs},{cap}}}{w}_{i}}{{\sum }_{i=1}^{{n}_{{cap}}}{w}_{i}}({B}_{t}-{\sum }_{f=1}^{u}{B}_{t,f})$$

where ${n}_{{fs},{cap}}$ is the number of assets captured in the subblock, ${n}_{{cap}}$ is total number of captured assets. Accordingly, the LP optimization for each captured subblock ${b}_{{fs},{cap}}$ is formulated to optimize its local performance metric, subject to this adjusted budget constraint:

$$\max \;{{\rm{LO}}}{{{\rm{S}}}}_{t,s}={\sum }_{i=1}^{{n}_{{fs},{cap}}}{w}_{i}{\mu }_{{it},s}$$

$$\min {{{\rm{Risk}}}}_{t,s}={\sum }_{i=1}^{{n}_{{fs},{cap}}}{{w}_{i}P}_{{it}}{L}_{{it}}$$

19 $${{\rm{subject\; to}}}\;(1-{\tau }_{t}){B}_{t,s}^{{\prime} }\le {\sum }_{i=1}^{{n}_{{fs},{cap}}}{c}_{{it},s}\le (1+{\tau }_{t}){B}_{t,s}^{{\prime} }$$

The Block-LP mutation does not rely on stochastic or heuristic ‘soft’ repairs. Instead, it systematically enforces feasibility through a mathematically rigorous hierarchical budget redistribution mechanism. Specifically, before executing the LP optimization on infeasible sub-blocks, the algorithm first locks in the exact budgets already consumed by the feasible sub-blocks (${B}_{t,f}$). The remaining annual budget is then redistributed across the selected captured sub-blocks according to Eq. 18, and the resulting reallocated local budget for each captured sub-block (${B}_{t,s}^{{\prime} }$) is imposed as a strict hard constraint in the corresponding LP subproblem. Because each local LP is solved exactly within its newly assigned budget interval, the recomposed sub-block decisions satisfy the adjusted local annual budget bounds in Eq. 19, while remaining consistent with the overall annual budget and the planning-horizon total budget constraint.

### Evolutionary search procedure, constraint handling

To improve the transparency of the NBGA implementation, this section supplements the preceding description of the framework architecture and block-based operators by outlining the full evolutionary search procedure. It clarifies how candidate solutions are initialized, evaluated, evolved, filtered, and reported, and further specifies the selection and configuration of key hyperparameters, the constraint-handling mechanism, and the penalty design.

#### Initialization of the solution population

Each chromosome represents a complete multiyear maintenance and renewal plan in the form of the action matrix $\{{j}_{{it}}\}$, covering all assets i=1,…,n and all years t=1,…,h. The initial population contains ${N}_{{pop}}$ chromosomes. Each chromosome is generated by randomly assigning admissible MRR actions to each asset-year cell, while satisfying the action-exclusion rule that only one action can be assigned to an asset in a given year. Because the annual budget, total budget, and service constraints couple decisions across assets and years, chromosomes generated at initialization need not be feasible. Instead, they are randomly generated to preserve diversity in the search space. Feasibility is then progressively improved during evolution through penalty-based fitness evaluation and the nested repair-and-refinement operators described in the previous section.

#### Fitness evaluation and penalty design

For any candidate chromosome x, the raw objective value is evaluated according to the system objective defined in the main formulation. For single-asset systems or systems with homogeneous performance measures, the objective is the network average level of service (avgLOS), corresponding to Eq. 1. For heterogeneous composite-asset systems, the objective is formulated in risk form, namely the time-averaged annual combined loss, corresponding to Eq. 2. Because these two cases differ in both scale and optimization direction, the implementation adopts a case-specific penalty design, rather than a single unified penalty formula.

For the single-asset road scalability tests, the fitness function is defined as

20 $$F\left(x\right)={avgLOS}(x)-P(x)$$

where ${avgLOS}(x)$ is the average annual network avgLOS over the planning horizon, and P(x) is the penalty term. In this case, the penalty follows a discrete counting mechanism. If the expenditure in year t falls outside the annual budget band $[\left(1-{\tau }_{t}\right){B}_{t},\,\left(1+{\tau }_{t}\right){B}_{t}]$, one annual budget violation is recorded and a fixed penalty ${P}_{a}=1$ is added. If the cumulative expenditure over the planning horizon exceeds the total budget limit, one additional fixed total-budget penalty ${P}_{T}=t$ is imposed. Thus,

21 $$P\left(x\right)={P}_{a}+{P}_{T}$$

Annual budget violations, therefore, enter the fitness as one fixed deduction per violating year, while total budget exceedance incurs a stronger additional penalty.

For the composite-asset risk case, the objective function is defined as

22 $$F\left(x\right)={Risk}+P(x)$$

where Risk denotes the average annual combined expected loss over the planning horizon. In this study, the yearly combined losses from the road and pipe systems are averaged over all planning years.

The penalty term P(x) is introduced to penalize annual budget-band violations, total-budget violations, and asset-class expenditure-share violations, and can be written as

23 $$P\left(x\right)={P}_{a}+{P}_{T}+{P}_{{share}}$$

The annual budget-band penalty ${P}_{a}$ meaning that whenever the expenditure in year t falls outside the annual budget band $[\left(1-{\tau }_{t}\right){B}_{t},\,\left(1+{\tau }_{t}\right){B}_{t}]$, an annual full-loss-scale penalty ${L}_{{annual}}$ is imposed. The total-budget penalty ${P}_{T}$ meaning that if the total expenditure over the full planning horizon exceeds the horizon-level budget limit, a full-horizon-loss-scale penalty ${L}_{{total}}$ is added. In addition, if the annual expenditure share assigned to roads or pipes falls below its prescribed lower bound, an asset-class share penalty is added:

24 $${P}_{{share}}\left(x\right)=\alpha \,{L}_{{annual}}{\sum }_{t=1}^{h}[\max \left(0,{\phi }_{R}-\hat{{\phi }_{R,t}}\right)+\max \left(0,{\phi }_{P}-\hat{{\phi }_{P,t}}\right)]$$

where ${\phi }_{R,t}$ and ${\phi }_{P,t}$ are the realized expenditure shares of roads and pipes in year t, ${\phi }_{R}$ and ${\phi }_{P}$ are their prescribed minimum shares, and α is the class-share penalty multiplier.

#### Parent selection and elite retention

At each generation, parent chromosomes are selected based on their current fitness values using tournament selection, with a tournament size of 3. To prevent high-quality solutions already discovered from being destroyed by subsequent stochastic crossover and mutation, an explicit elitist-retention strategy is adopted. Specifically, ${N}_{{pop}}-{N}_{{hof}}$ individuals are first selected from the current population to enter the reproduction stage, where ${N}_{{hof}}$ denotes the number of elite individuals retained in the Hall-of-Fame. These elite chromosomes are preserved before crossover and mutation are applied to the remaining population. This mechanism stabilizes convergence and ensures that improvements discovered in earlier generations are not destroyed by subsequent stochastic operations. In the current implementation, the Hall-of-Fame size is set to 10% of the population size.

#### Offspring generation under the NBGA operators

The non-elite portion of the next generation is produced by the NBGA search operators. First, APS crossover is applied at the annual-plan level by randomly selecting a year and swapping the corresponding annual plans between two parent chromosomes. Next, for the selected planning year, the asset set is decomposed through the blocking and nesting hierarchy. Depending on the nesting depth, MRR mutation is first applied to the parent block, after which block crossover propagates compact decision structures among sibling sub-blocks. At the end level, Block-LP mutation is used as a targeted refinement step for sub-blocks identified as infeasible or weak in improvement potential. The LP refinement is therefore embedded within the evolutionary search as a structured local search operator rather than as a stand-alone deterministic optimizer.

#### Termination criteria, convergence curves, and final returned solutions

The evolutionary process continues until the maximum number of generations is reached. In the current implementations, the maximum number of generations is fixed at 30 in both cases. At each generation, only offspring with invalid fitness values are reevaluated, after which generation-level statistics are recorded. Final results are not reported directly from the full population. Instead, they are obtained from the elite solution set retained in the Hall of Fame, and the best-ranked elite individual is typically taken as the final returned solution of an independent run.

### Worst-first rolling-horizon (WF)

To rigorously evaluate the benefits of the multi-year global spatiotemporal coordination achieved by NBGA, we implemented a worst-first rolling-horizon strategy as a representative non-GA baseline. This greedy heuristic is widely used in conventional infrastructure management practice due to its computational simplicity, as it decomposes the h-year optimization into independent, single-year budget allocation steps based strictly on immediate asset conditions.

Unlike NBGA, which optimizes the entire multi-year decision matrix simultaneously, the Worst-First rolling-horizon strategy allocates budgets one year at a time using a strict prioritization rule. The sequential steps are as follows:

1. condition ranking: at the beginning of year t, all assets in the inventory are evaluated and ranked in ascending order based on their current physical condition (i.e., Pavement Quality Index, ${{PQI}}_{i,t}$).
2. greedy allocation: the algorithm selects the worst-performing asset at the top of the ranked list and assigns the appropriate Maintenance, Rehabilitation, and Replacement (MRR) action. The cost of this action is deducted from the available annual budget ${B}_{t}$.
3. budget exhaustion: step 2 is repeated sequentially for the next worst assets in the ranked list. The allocation for year t terminates when the remaining annual budget is insufficient to fund any further MRR actions without violating the tolerance bound $\left(1+{\tau }_{t}\right){B}_{t}$.
4. state transition: the selected MRR actions are applied to update the asset conditions. Deterministic deterioration models are then applied to all assets to predict their starting conditions for the subsequent year (t+1). The process increments to the next year and repeats until year h is reached.

## Supplementary information

Supplementary Information for A nested-block scalable framework for efficient and equitable urban infrastructure management

## Data Availability

The dataset used in this study can be found at the following URL: https://github.com/urinfra/nbga-urban-infrastructure-release.

## Code availability

The code for NBGA can be found at the following URL: https://github.com/urinfra/nbga-urban-infrastructure-release.
