# Supplementary material for: A nested-block scalable framework for efficient and equitable urban infrastructure management
Source: Commun Eng. 2026 Jul 13;5:186. doi: 10.1038/s44172-026-00729-x (PMC13737207; doi:10.1038/s44172-026-00729-x)
Supplement: Supplementary file 1 [file 44172_2026_729_MOESM1_ESM.pdf]

Supplementary Information for

A nested-block scalable framework for efficient and equitable urban infrastructure management

Peiyuan Lin[1]*†, Xiangwei Yu[1]†, Tong Liu[1], Xun Yuan[1], Chuhao Long[1], Yang Chen[1], Haoyi Li[1], Jun Xiao[1], Baosong Ma[1], Linchong Huang[1]*, Xian-Xun Yuan[2]*

[1] School of Civil Engineering, Sun Yat-Sen University, Southern Marine Science and Engineering Guangdong Laboratory, State Key Laboratory for Tunnel Engineering, Zhuhai 519082, China.

[2] Department of Civil Engineering, Toronto Metropolitan University, 350 Victoria St., Toronto, ON M5B 2K3, Canada.

*Corresponding author. Email: linpy23@mail.sysu.edu.cn (P.-Y.L.); hlinch@mail.sysu.edu.cn (L.-C.H.); arnold.yuan@torontomu.ca (X.-X.Y.).

†These authors contributed equally to this work.

The PDF file includes:

Supplementary Text

Nomenclature

Figs. S1 to S18

Tables S1 to S3

Supplementary Text

Operational mapping of nesting strategies in real-world

Figure S2 conceptually illustrates the real-world operational logic of the blocking-nesting strategies used in this study. As shown in the figure, multiple asset classes (A, B, and C) are first aggregated into a unified asset inventory. These asset classes may represent different types of infrastructure assets, such as roads, pipes, or other urban assets. Under the NBGA framework, this inventory is then organized into hierarchical planning units, including higher-level Blocks (for example, 1, 2, and 3) and lower-level Sub-blocks (for example, 1.1, 1.2, 1.3, 2.1, 2.2, 3.1, and 3.2).

A two-level strategy corresponds to an asset inventory–block hierarchy. In this case, the unified asset inventory is partitioned into a set of higher-level blocks, such as blocks 1, 2, and 3 in Fig. S2. Operationally, these blocks can represent governance- and implementation-oriented planning units, such as administrative districts, managerial zones, spatial partitions, or major project areas. For example, in a metropolitan setting, a two-level structure may correspond to a city–district planning hierarchy, in which overall coordination is maintained at the city level while implementation is organized within district-level units.

A three-level strategy corresponds to an asset inventory–block–sub-block hierarchy. In addition to the higher-level blocks, each block is further divided into smaller sub-blocks, such as 1.1–1.3 under block 1, 2.1–2.2 under block 2, and 3.1–3.2 under block 3. In operational terms, this represents a more fine-grained planning hierarchy, in which the lower-level sub-blocks may correspond to neighborhood-scale units, corridor-scale units, local project packages, or other localized implementation units. For example, in a city-scale context, this may resemble a city–district–street or city–district–neighborhood management structure. This additional nesting depth improves local resolution, but it also increases coordination complexity.

The hierarchy shown in Fig. S2 should be understood as a conceptual mapping rather than a fixed one-to-one correspondence. For example, in a regional-scale application, the top-level asset inventory may represent the entire regional asset portfolio, the blocks may correspond to different cities or major managerial units, and the sub-blocks may further correspond to urban districts or localized implementation units. Similarly, from an asset-organization perspective, the same hierarchy may also represent different levels of operational classification and implementation grouping.

Therefore, the differences among single-level, two-level, and three-level strategies should not be understood merely as differences in numerical partition depth. Rather, they represent different planning hierarchies with different degrees of management centralization, local implementation resolution, and coordination complexity. In general, a single-level strategy emphasizes stronger centralized coordination but may mask local heterogeneity; a two-level strategy typically provides a better balance between system-level coordination and localized implementation; and a three-level strategy offers the highest local resolution, but often requires more complex cross-level coordination.

Unified efficiency metric

Direct comparison across heterogeneous indicators requires normalization. For strategy i

$$S_{avgLOS}(i) = \frac{avgLOS(i) - avgLOS(min)}{avgLOS(max) - avgLOS(min)} \quad \text{(S1)}$$

$$C_{time}(i) = \frac{Time(i) - Time(min)}{Time(max) - Time(min)} \quad \text{(S2)}$$

$$C_{spend}(i) = \frac{Spend(i) - Spend(min)}{Spend(max) - Spend(min)} \quad \text{(S3)}$$

Define the unified Efficiency Score

$$Escore(i) = \frac{S_{avgLOS}(i)}{\omega_{time} C_{time}(i) + \omega_{spend} C_{spend}(i)} \quad \text{(S4)}$$

Here,ω_{time} and ω_{spend} are non-negative weights representing the relative importance of time and expenditure, satisfying ω_{time}+ω_{spend}=1. For the analysis in the main text, ω_{time}=0.6, ω_{spend}=0.4.

Risk and equity metrics

To normalize network-wide risk across portfolios of different total values, we define the Average Annual Risk Percentage ($R_{avg\%,t}$). This metric represents the total expected monetary loss in a given year t as a percentage of the total maximum possible loss (i.e., the replacement cost of all assets in the network).

$$R_{avg\%,t} = \frac{R_{network,t}}{L_{total}} = \frac{\sum_{i=1}^{n} (P_{i,t} \cdot L_i)}{\sum_{i=1}^{n} L_i} \quad \text{(S5)}$$

where, n is the total number of assets in the network; $P_{i,t}$ is the calculated probability of failure (PoF) for asset i in year t; L_i is the event loss, or consequence of failure, for asset i (e.g., replacement cost and/or social costs); L_{total} is the total maximum failure loss for the entire network (sum of all individual event losses).

To quantify the inequality in the distribution of risk across assets, we use the Gini coefficient (G_t). This metric measures the statistical dispersion of risk values among all assets in year t. The Gini coefficient is calculated based on the relative mean absolute difference between all pairs of asset risks:

$$G_t = \frac{\sum_{i=1}^{n} \sum_{j=1}^{n} |R_{it} - R_{jt}|}{2n \sum_{k=1}^{n} R_{kt}} = \frac{\sum_{i=1}^{n} \sum_{j=1}^{n} |(P_{it} \cdot L_{it}) - (P_{jt} \cdot L_{jt})|}{2n^2 \cdot \overline{R_t}} \tag{S6}$$

where, R_{it} and R_{jt} are the risk values for assets i and j in year t; n is the total number of assets; $\overline{R_t}$ is the mean risk value across all assets in year t. A value of $G_t = 0$ indicates perfect equity (all assets carry the exact same risk), while a value approaching $G_t = 1$ indicates perfect inequity (all network risk is concentrated in a single asset).

To measure the model's active intervention in high-priority, vulnerable assets, we define Equity Coverage (EC_t). This metric quantifies the proportion of 'neglected' assets that receive an intervention in a given year t.

$$EC_t = \frac{|A_{intervened,t}|}{|A_{neglected,t}|} \tag{S7}$$

where $A_{nelegected,t}$ is the set of 'neglected' assets in year t. Based on the analysis in Fig. 4c, these are defined as assets i that are simultaneously in a high failure probability state and have a high event loss, i.e., $A_{nelegected,t} = \{i | P_{i,t} \geq P_{threshold}\}$. $A_{intervened,t}$ is the subset of neglected assets that receive a maintenance or renewal intervention in year t ($A_{intervened,t} \subseteq A_{nelegected,t}$). $|\cdot|$ denotes the count (cardinality) of the set.

<u>Case background</u>

To evaluate the proposed method performance in real-world and large-scale scenarios, we constructed three single-asset road network cases. Building upon these, we further established a corresponding dual-asset (road-pipe) composite case. This setup is used to validate the model's effectiveness in a multi-asset context, analyze the sensitivity of outcomes to budget and network scale, and discuss the equity and trustworthiness crises in urban-scale infrastructure networks and their control strategies. All cases adhere to a unified mathematical framework, encompassing the 'Mathematical Formulation and Constraints' (cf. Eqs. 1–4), the 'Deterioration-Maintenance-Failure Process' (Eqs. 5–10), and the 'Blocked and Nested Optimization Framework' (Eqs. 11–19), and employ uniform performance metrics (Eqs. S1–S4).

In the single-asset cases, pavement performance is measured by the Pavement Quality Index (PQI, where 0 is the worst and 10 is the best), and the network level of service (avgLOS) is represented by a weighted average (weighted by segment length). The annual PQI evolution for each road segment is dynamically updated in two stages: natural deterioration and instantaneous improvement from MRR actions. The natural deterioration process employs an accelerated decay function ($S = 10, k = 0.1, \varepsilon = 1 \times 10^{-9}$). The four MRR action types are: no action, maintenance, rehabilitation, and reconstruction, with instantaneous improvement magnitudes (ζ) set to 0, 0.8, 3, and 10, respectively. Maintenance costs are set to be heterogeneous across segments, based on real or statistical costs related to materials and location. Case 1 uses a real network from a North American city with 13,610 segments and 4,417 km total length; initial avgLOS = 5.86. The planning horizon is 5 years with a total budget of 150 million; the annual baseline is 30 million with ±20% tolerance. Fig. S3 reports the distributions of initial PQI and segment-level costs for the four MRR actions. Case 2 is a virtual city-scale network expanded to 100,000 segments by duplicating Case 1 seven times (95,270 segments) and randomly adding 4,730 segments; initial avgLOS = 5.85. The planning horizon is 10 years with a total budget of 3000million, with all other settings aligned with Case 1; Fig. S3 also presents PQI and cost distributions for Case 2. Case 3 further constructs an ultra-large network of 1,000,000 segments by duplicating the original network 73 times (993,530 segments) and adding 6,470 randomly selected segments; initial avgLOS = 5.86. The annual budget is proportionally set to 30 billion.

To rigorously demonstrate the proposed method's performance and eliminate any potential symmetry biases introduced by the pure duplication in Case 2, we further constructed three variant datasets (Cases 2-1, 2-2, and 2-3) by applying random perturbations to the initial asset conditions (PQI) and maintenance costs (C_{ij}). To ensure full reproducibility, the specific mathematical constructions are detailed below, where $u(a, b)$ denotes a uniform distribution and $\mathcal{N}(\mu, \sigma^2)$ denotes a normal distribution. All perturbed PQI values are strictly bounded within the valid range [0, 10], and costs are enforced to be positive.

Case 2-1 (Uniform Micro-Perturbation): Introduces a ±5% uniform noise to simulate baseline observational errors, initial avgLOS = 5.61. The parameters are generated as

$$C_{ij} = C_{ij}^{base}(1 + \Delta_C); \quad PQI = PQI^{base}\left(1 + \Delta_{PQI}\right) \tag{S8}$$

where both Δ_C and $\Delta_{PQI} \sim u(-0.05, 0.05)$.

Case 2-2 (Gaussian Independent Perturbation): Applies normal distribution variance to simulate natural variance and long-tail asset deterioration, initial avgLOS = 5.61. The parameters are updated as

$$C_{ij} = C_{ij}^{base} \times max(\epsilon_C, 0.1); \quad \epsilon_C \sim \mathcal{N}(1, 0.1^2) \tag{S9}$$

$$PQI = PQI^{base} + \epsilon_{PQI}; \quad \epsilon_{PQI} \sim \mathcal{N}(0, 0.5^2) \tag{S10}$$

Case 2-3 (Macro-Geographic Heterogeneity + Micro-Perturbation): Simulates inflation or extreme climate impacts in different administrative zones. The duplicated network is divided into independent geo-blocks, initial avgLOS = 5.57. Each block is assigned macro-scaling factor $\Psi_C^{geo} \sim u(0.8,1.3)$ and $\Psi_{PQI}^{geo} \sim u(0.85,1.15)$. The asset properties within geo-blocks are then generated by

$$C_{ij} = C_{ij}^{base} \times \Psi_C^{geo} \times (1 + \Delta_C); \quad \Delta_C \sim u(-0.05,0.05) \tag{S11}$$

$$PQI = PQI^{base} \times \Psi_{PQI}^{geo} + \epsilon_{PQI}; \quad \epsilon_{PQI} \sim \mathcal{N}(0, 0.3^2) \tag{S12}$$

Fig. S4 presents the detailed distributions of initial PQI and maintenance costs under these three perturbed scenarios.

To comprehensively study investment intensity in urban evolution and the associated issues of equity and trustworthiness, a municipal water pipe network is introduced as a second asset layer upon the road networks to create the dual-asset composite case. To investigate the impact of different asset compositions and scales, this composite case is configured in three categories: 100,000 road segments + 300,000 pipe segments, 200,000 road segments + 200,000 pipe segments, and 300,000 road segments + 100,000 pipe segments. The objectives and constraints for all combinations still follow Eqs. 1–4. In this dual-asset model, the failure risk for roads is estimated using a Gamma process, while the annual failure process for pipe assets is modeled using the NHPP (Eqs. 7–10). Pipe maintenance actions include patching, lining, and replacement. Their effects on the annual expected number of failures are characterized by "intensity scaling and age rejuvenation." Specifically, patching scales the expected annual failures by a factor of 0.8; lining provides a 0.6 age rejuvenation and simultaneously scales the intensity by 0.6. Action costs are heterogeneously set for each pipe segment (correlated with diameter, depth of cover, and length). The dual-asset system shares a single annual budget envelope with an allowed bandwidth (Eq. 3). Spatially, both asset types are organized within a unified blocked/nested optimization structure to facilitate governance-oriented and block-level cross-asset coordination (see Eqs. 11–19). These

blocks are planning units defined by operational context, such as jurisdiction, spatial grouping, or corridor-like project areas, rather than graph-topological subnetworks.

Uniform and non-uniform partitioning tests on perturbed asset datasets

To solve the large-scale perturbed datasets (Cases 2-1, 2-2, and 2-3), the 100,000-segment network is optimized using a dual-level nested framework: 10 major regional blocks, each subdivided into 5 minor subblocks, yielding 50 localized subproblems. To comprehensively evaluate NBGA robustness in handling diverse asset datasets, we implemented both uniform and non-uniform partitioning tests across all three perturbed scenarios.

1. Uniform blocking: the network is divided evenly, each of the 10 major blocks receives 10% of the total network (10,000 segments), and each of the 5 minor subgroups receives exactly 20% of its parent block (2,000 segments). Consequently, all 50 subproblems handle 2,000 segments.

2. Dual-level Tiered Non-uniform Grouping: Real-world administrative zones inherently exhibit skewed spatial distributions (e.g., hyper-dense metropolitan cores versus sparse rural outskirts). To accurately reflect this administrative reality, we applied a Pareto-like deterministic scaling to the block sizes. At the macro-level, the 10 major blocks are scaled to proportions of 25%, 15%, 15%, 10%, 10%, 5%, 5%, 5%, 5%, and 5%. At the micro-level, the 5 subgroups within each major block are further skewed to 40%, 25%, 15%, 10%, and 10%. Under this non-uniform configuration, the largest subproblem encompasses 10,000 segments (100,000 × 25% × 40%), while the smallest handles only 500 segments (100,000 × 5% × 10%), creating a drastic 20-fold computational discrepancy.

The results of these stress tests conclusively demonstrate the exceptional robustness and adaptability of the NBGA framework. As illustrated in Fig. S11, the convergence behaviors of NBGA are remarkably consistent across both uniform and non-uniform blocking schemes. For all three highly perturbed datasets (Cases 2-1, 2-2, and 2-3), the algorithm rapidly escapes the initial stochastic state and achieves steady-state convergence within 15 generations (Figs. S11a and S11b). The scatter plots of the final solutions (Figs. S11c and S11d) further confirm that the severe structural asymmetry introduced by the non-uniform partitioning does not degrade the optimization quality; the framework consistently discovers tight clusters of cost-effective, high-LOS solutions.

Furthermore, Fig. S12 visualizes the hierarchical regulation of asset conditions from the macro-level (major blocks) to the micro-level (subblocks). Under the uniform scheme (Figs. S12a-

c), the avgLOS is tightly balanced across all partitions. More importantly, under the stress of the dual-level non-uniform scheme (Figs. S12d-f), where subproblem sizes vary by up to 20 times, NBGA successfully prevents localized "run-to-failure" scenarios. While the smaller subblocks naturally exhibit a slightly wider variance in avgLOS due to their limited asset pools, the overarching condition of the major blocks remains highly stable and concentrated.

Nomenclature

n	Total number of assets in the network
h	Planning horizon (number of years)
i	Index for asset ($i = 1, \ldots, n$)
t	Index for year ($t = 1, \ldots, h$)
j_{it}	Do nothing, Maintenance, Rehabilitation, or Reconstruction (MRR) action for asset *i* in year *t*
$A_{neglected,t}$	Set of 'neglected' assets in year *t*
$A_{intervened,t}$	Subset of neglected assets that receive an intervention in year *t*
μ_{it}	Condition of asset *i* in year *t*
P_{it}	Probability of Failure (PoF) for asset *i* in year *t*
w_i	Weight of asset *i* (e.g., length or importance)
L_{it}	Direct and indirect consequences of failure (Event Loss) for asset *i*
c_{it}	Cost of the MRR action j_{it}
B_t	Designated budget for year *t*
τ_t	Budget tolerance for year *t*
B_{total}	Total budget for the planning horizon
s	Scale parameter for natural deterioration
k	Rate parameter for natural deterioration
ε	Near-zero threshold to prevent condition from becoming negative/zero
ζ	Instantaneous improvement in condition after MRR action
$\mu_{it(\max)}$	Maximum possible condition value a segment can achieve
X	Cumulative damage over a one-year period (Random Variable)
α	Shape parameter for Gamma distribution
θ	Scale parameter for Gamma distribution
C_{fail}	Predefined critical threshold for failure
δ_j	Improvement amount in condition for repair-type actions
d	Damage threshold to cause failure within the year
$\Lambda_i(t)$	Cumulative intensity function for Non-Homogeneous Poisson Process (NHPP)
β	Shape parameter for NHPP (failure rate trend)
M_i	Scale factor for NHPP reflecting physical attributes
t_y	Asset service age
ρ_{patch}	Repair factor for minimal repair

$\kappa_{linling}$	Rejuvenation factor for imperfect repair
ρ_{lin}	Repair factor for imperfect repair
b_f	Block (parent level)
b_{fs}	Sub-block (offspring level)
IPV_s	Improvement Potential Value for a sub-block
K	Target number of sub-blocks to optimize (Capture phase)
P_s	Base selection probability for a sub-block
ρ_{min}, ρ_{max}	Lower and upper bounds for selection probability
$B_{t,s}$	Budget allocated to sub-block b_{fs}
$B'_{t,s}$	Adjusted budget for sub-block in LP Mutation
n_b	Number of total blocks or subblocks
ρ_{pb}	Proportion of assets in a parent block
n_{pb}	Number of assets in a parent block
ρ_{ob}	Proportion of assets in a subblock
n_{ob}	Number of assets in a subblock
avgLOS	Average Network Level of Service
N_{pop}	Population size
N_{hof}	The number of elite individuals
P_a	Annual-budget penalty
P_T	Total-budget penalty
P_{share}	Asset minimum annual expenditure shares penalty
$\widehat{\phi_{R,t}}$	The realized expenditure shares of roads in year t
$\widehat{\phi_{P,t}}$	The realized expenditure shares of pipes in year t
ϕ_R	The minimum expenditure shares of roads in year t
ϕ_P	The minimum expenditure shares of pipes in year t
$S_{avgLOS}(i)$	Normalized avgLOS for strategy *i*
$C_{time}(i)$	Normalized computation time for strategy *i*
$C_{spend}(i)$	Normalized expenditure for strategy *i*
$E_{score}(i)$	Unified Efficiency Score for strategy *i*
ω_{time}	Weight for relative importance of time
ω_{spend}	Weight for relative importance of expenditure
$R_{avg\%,t}$	Average Annual Risk Percentage
L_{total}	Total maximum failure loss for the entire network
G_t	Gini coefficient for risk distribution in year *t*
EC_t	Equity Coverage in year *t*

Fig. S1.

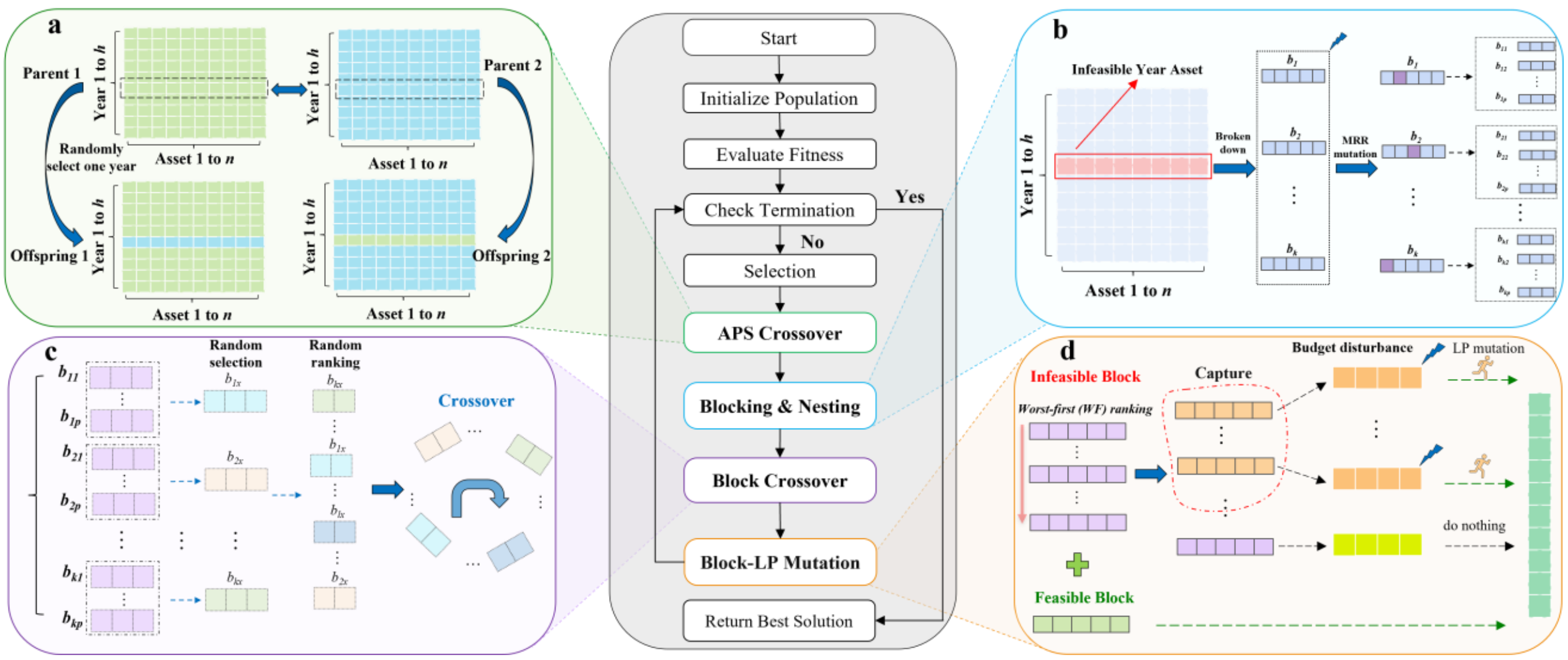

Fig. S1. NBGA framework process. (a) Annual-plan-swapping (APS) crossover. (b) Blocking and nesting method. (c) Block crossover method. (d) Block-LP mutation method.

Fig. S2.

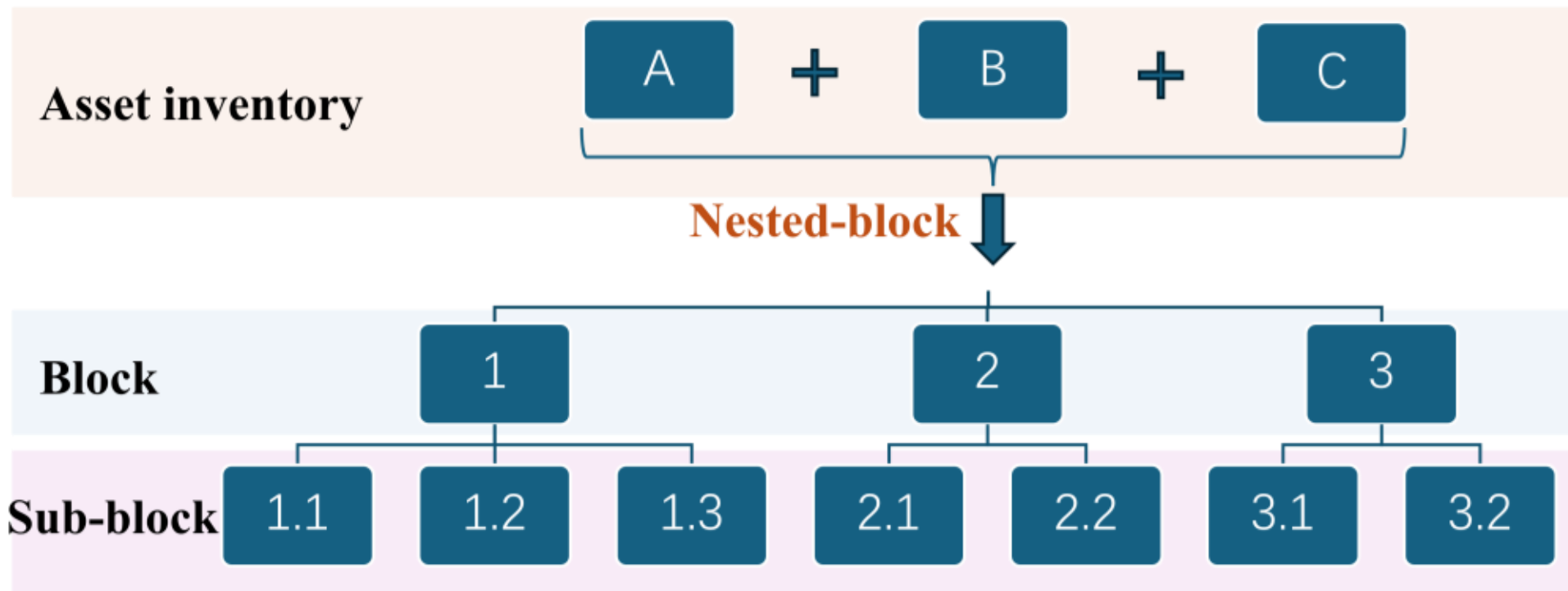

Fig. S2. Hierarchical decomposition of a multi-class asset inventory into block and sub-block planning units.

Fig. S3.

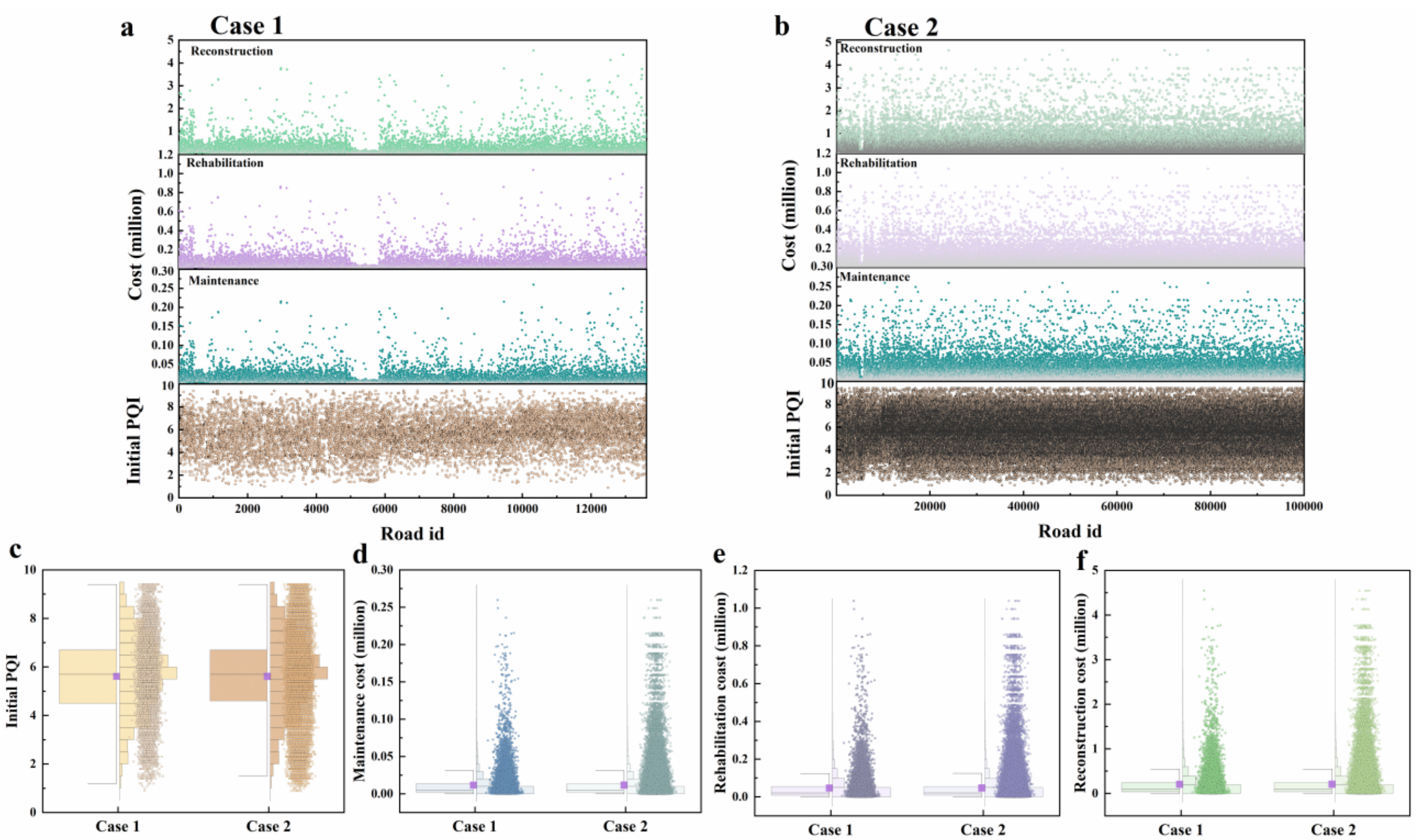

Fig. S3. MRR action expenditure and the initial PQI distribution of the network. (a) The initial PQI of each section and the cost corresponding to the MRR action in Case 1. (b) The initial PQI of each section and the cost corresponding to the MRR action in Case 2. (c) The distribution of initial PQI. (d) The distribution of maintenance cost. (e) The distribution of rehabilitation cost. (f) The distribution of reconstruction cost.

Fig. S4.

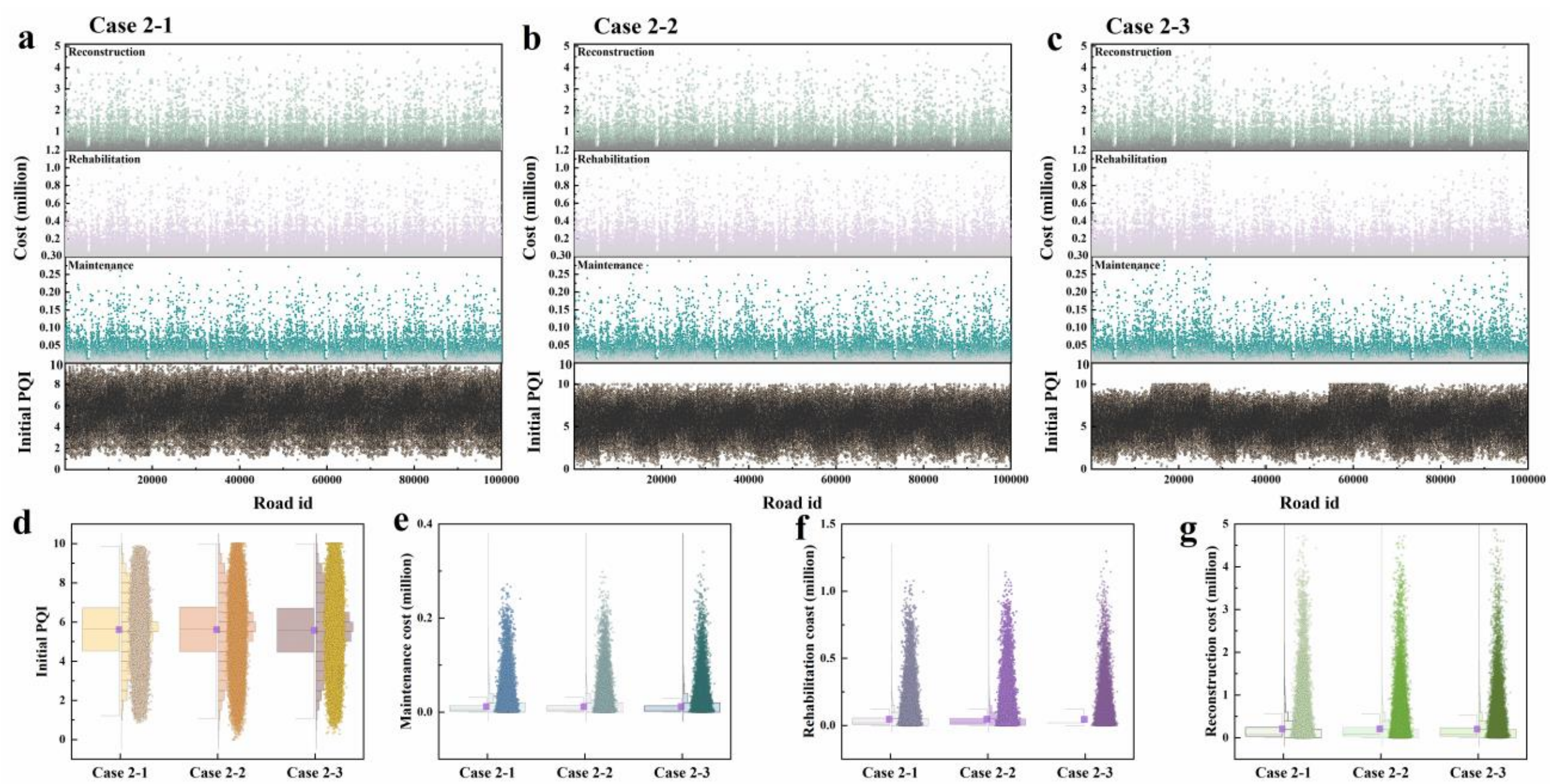

Fig. S4. MRR action expenditure and the initial PQI distribution of the network. (a) The initial PQI of each section and the cost corresponding to the MRR action in Case 2-1. (b) The initial PQI of each section and the cost corresponding to the MRR action in Case 2-2. (c) The initial PQI of each section and the cost corresponding to the MRR action in Case 2-3. (d) The distribution of initial PQI. (e) The distribution of maintenance cost. (f) The distribution of rehabilitation cost. (g) The distribution of reconstruction cost.

Fig. S5.

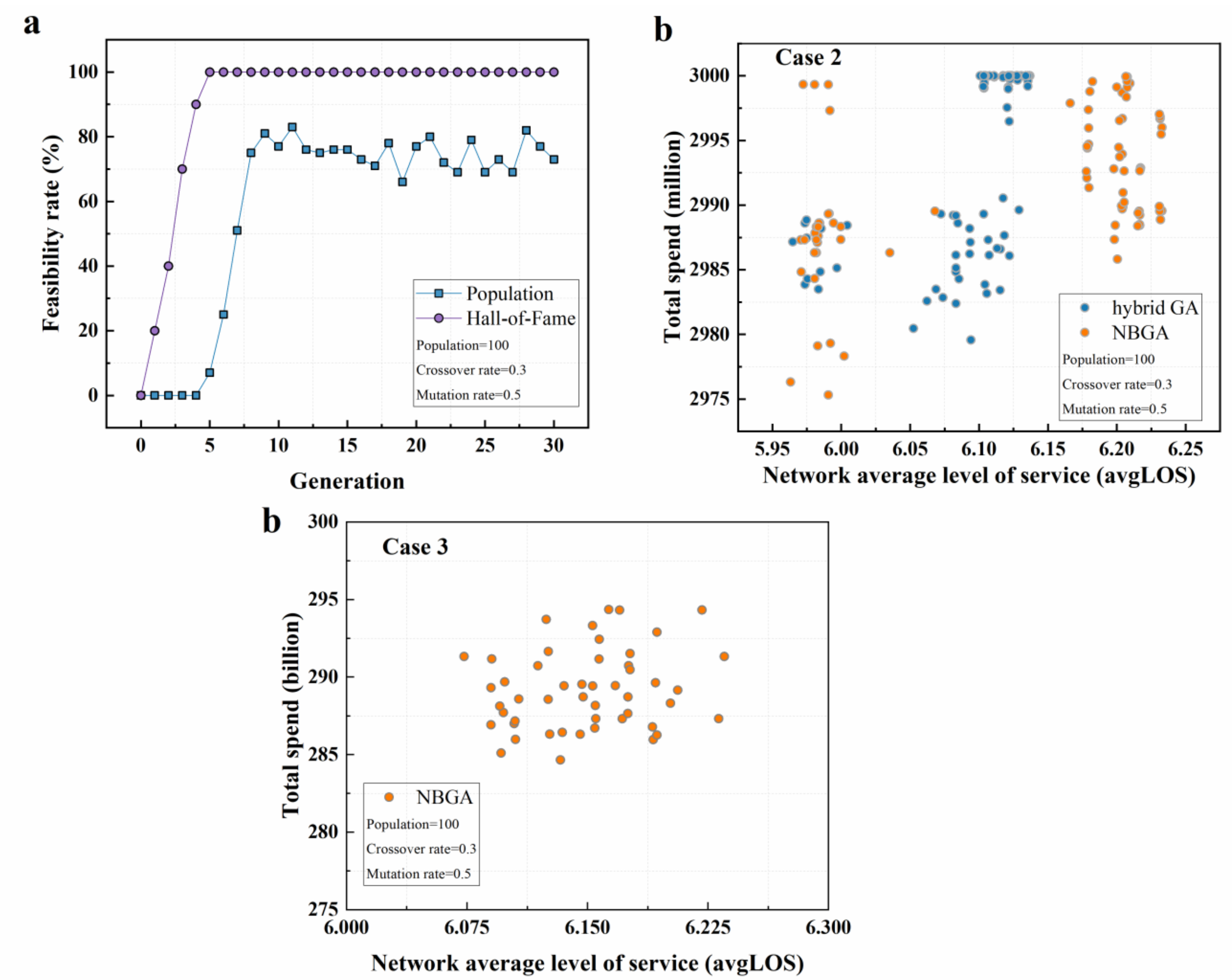

Fig. S5. Feasibility evolution and optimization robustness for large-scale asset inventories. (a) Generational evolution of the solution feasibility rate, comparing the overall population and the elite 'Hall-of-Fame' (HOF) archive. (b) Scatter plot of final solutions (total spend vs. average LOS) across independent parallel runs for Case 2 (100,000 assets). (c) Scatter plot of final NBGA solutions across independent parallel runs for Case 3 (1,000,000 assets).

Fig. S6.

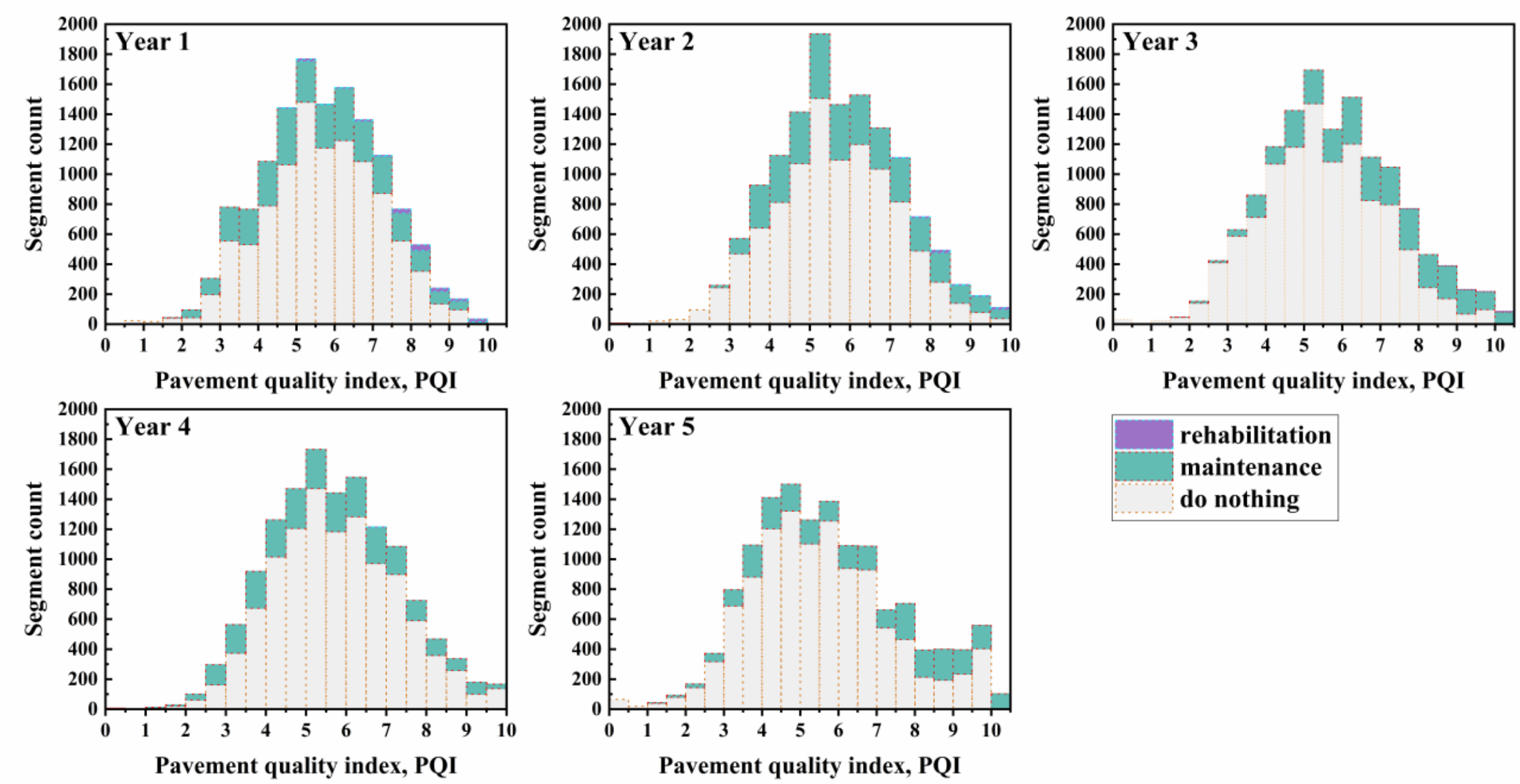

Fig. S6. Evolution of Pavement Quality Index (PQI) distribution and applied maintenance decisions for Case 1 over the 5-year planning horizon with NBGA. The five panels show the network-wide histogram of road segment counts across 10 PQI bins for Year 1 through Year 5. Within each bin, the stacked bars delineate the maintenance actions applied in that year: 'rehabilitation' (purple), 'maintenance' (teal), and 'do nothing' (dotted line/light fill). The figure illustrates how the optimization framework allocates interventions annually to manage the network's condition, mitigating natural deterioration and maintaining a stable quality distribution.

Fig. S7.

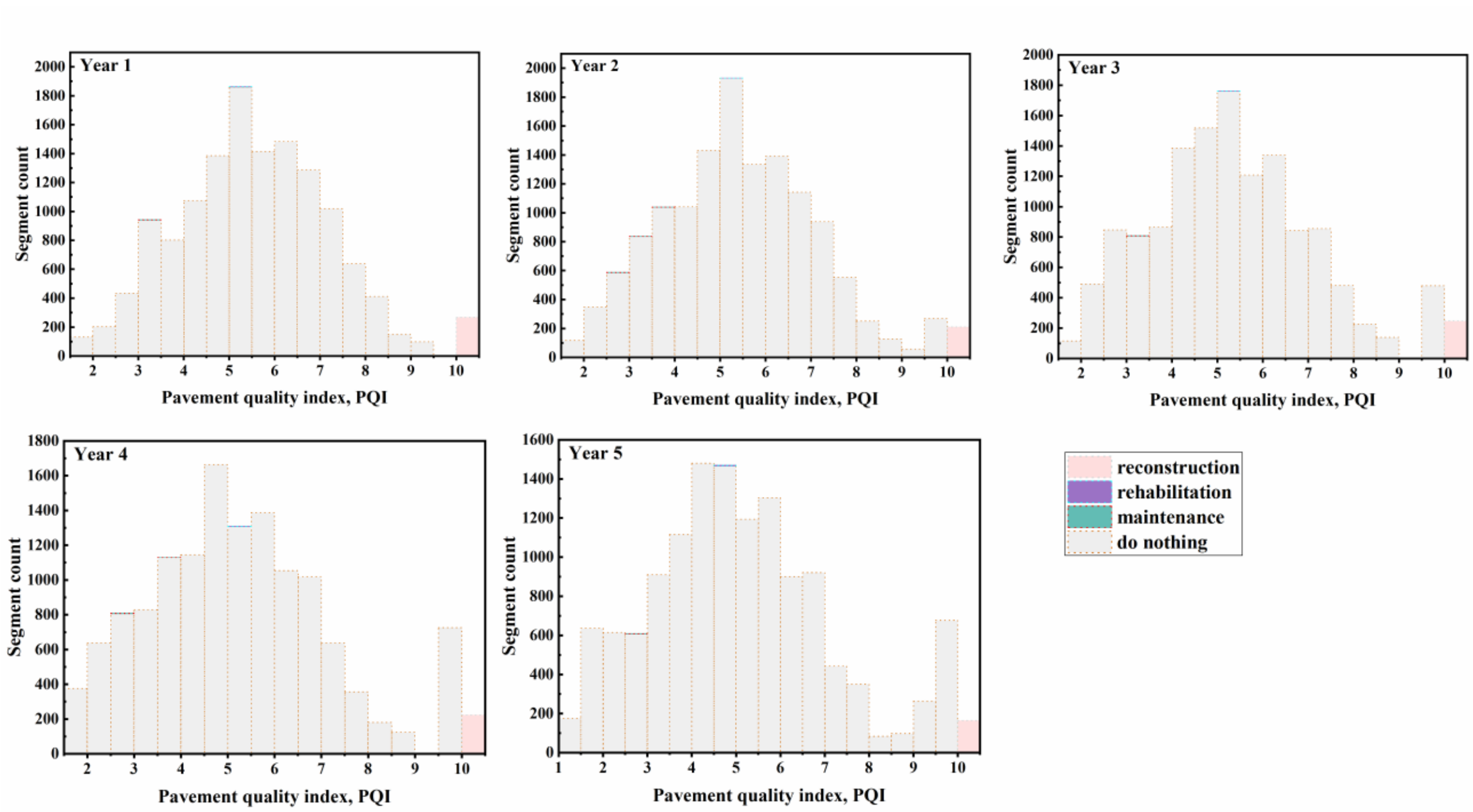

Fig. S7. Evolution of Pavement Quality Index (PQI) distribution and applied maintenance decisions for Case 1 over the 5-year planning horizon with WF. The five panels show the network-wide histogram of road segment counts across 10 PQI bins for Year 1 through Year 5. The figure illustrates how the optimization framework allocates interventions annually to manage the network's condition, mitigating natural deterioration and maintaining a stable quality distribution.

Fig. S8.

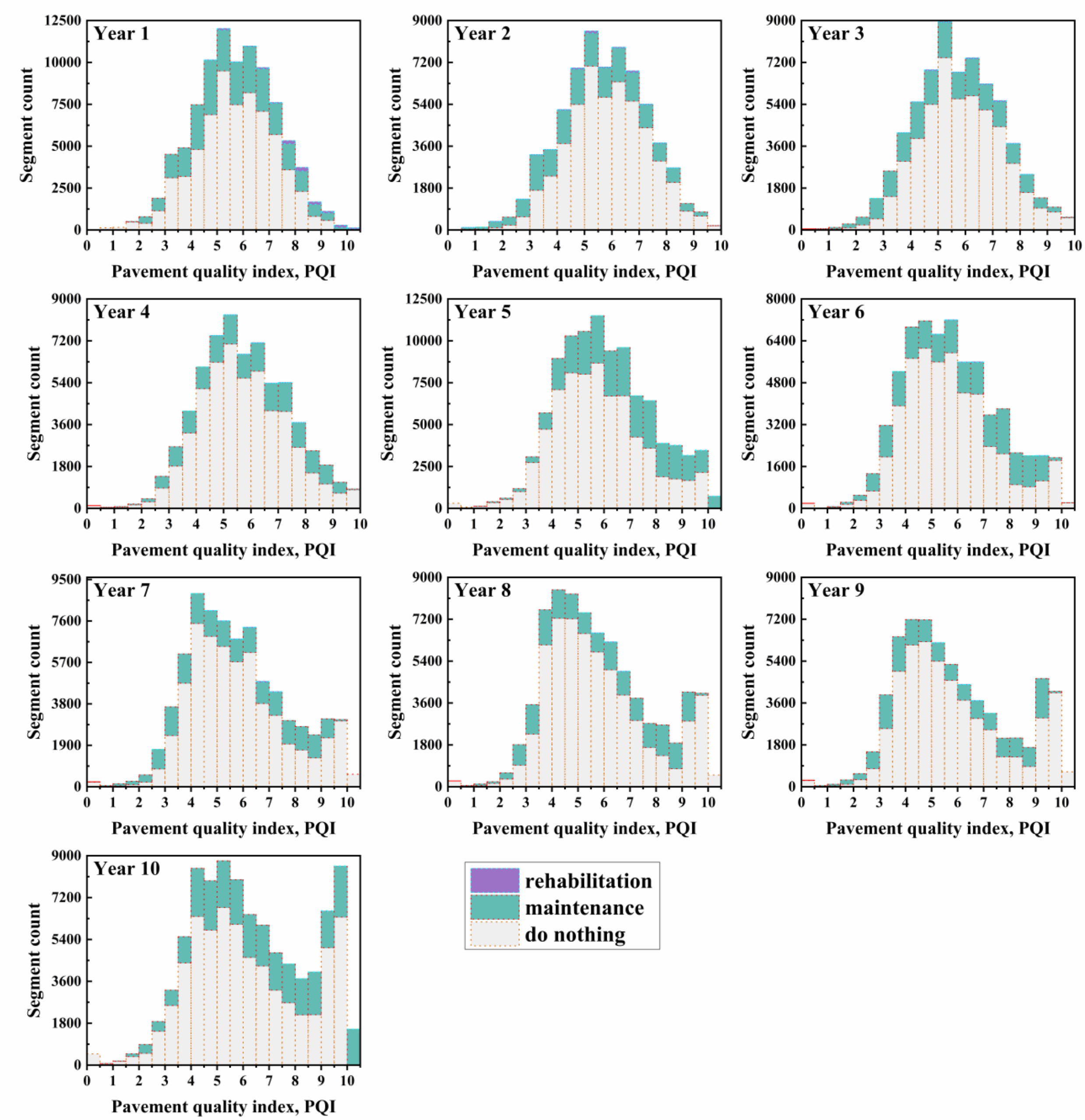

Fig. S8. Evolution of Pavement Quality Index (PQI) distribution and applied maintenance decisions for Case 2 over the 10-year planning horizon. The ten panels show the network-wide histogram of road segment counts across 10 PQI bins for each year (Year 1 to Year 10). Within each bin, the stacked bars delineate the maintenance actions applied in that year: 'rehabilitation' (purple), 'maintenance' (teal), and 'do nothing' (dotted line/light fill). The figure demonstrates the framework's ability to dynamically manage the network condition over a long-term horizon, applying interventions to counteract deterioration and maintain a relatively stable, high-quality PQI distribution.

Fig. S9.

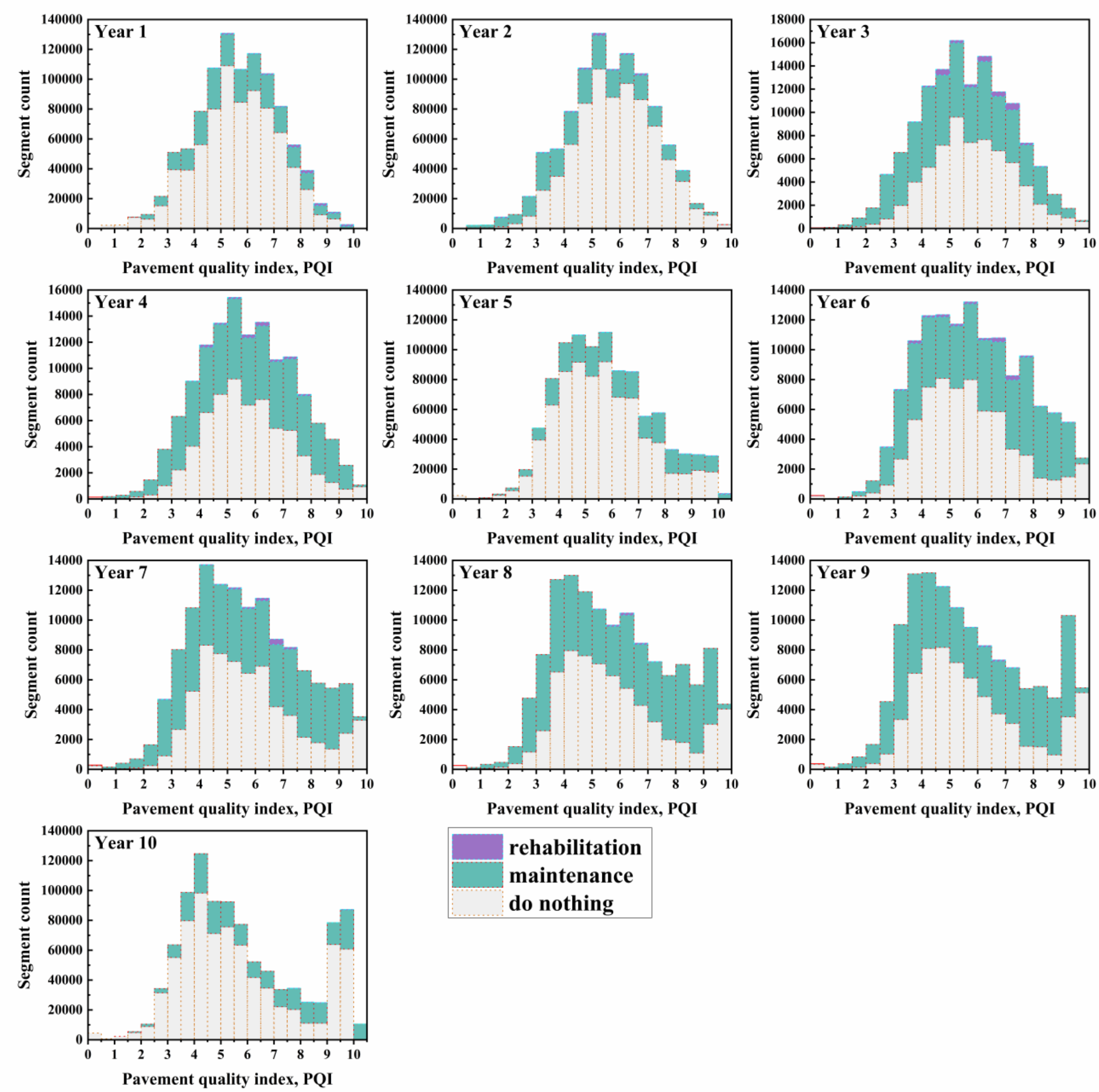

Fig. S9. Evolution of Pavement Quality Index (PQI) distribution and applied maintenance decisions for Case 3 (Million-Asset Scale) over the 10-year planning horizon. The ten panels show the network-wide histogram of road segment counts across 10 PQI bins for each year (Year 1 to Year 10). Within each bin, the stacked bars delineate the maintenance actions applied in that year: 'rehabilitation' (purple), 'maintenance' (teal), and 'do nothing' (dotted line/light fill). The figure demonstrates the framework's scalability, successfully managing the condition of a million-asset network to maintain a stable, normally-distributed PQI profile over the long-term horizon.

Fig. S10.

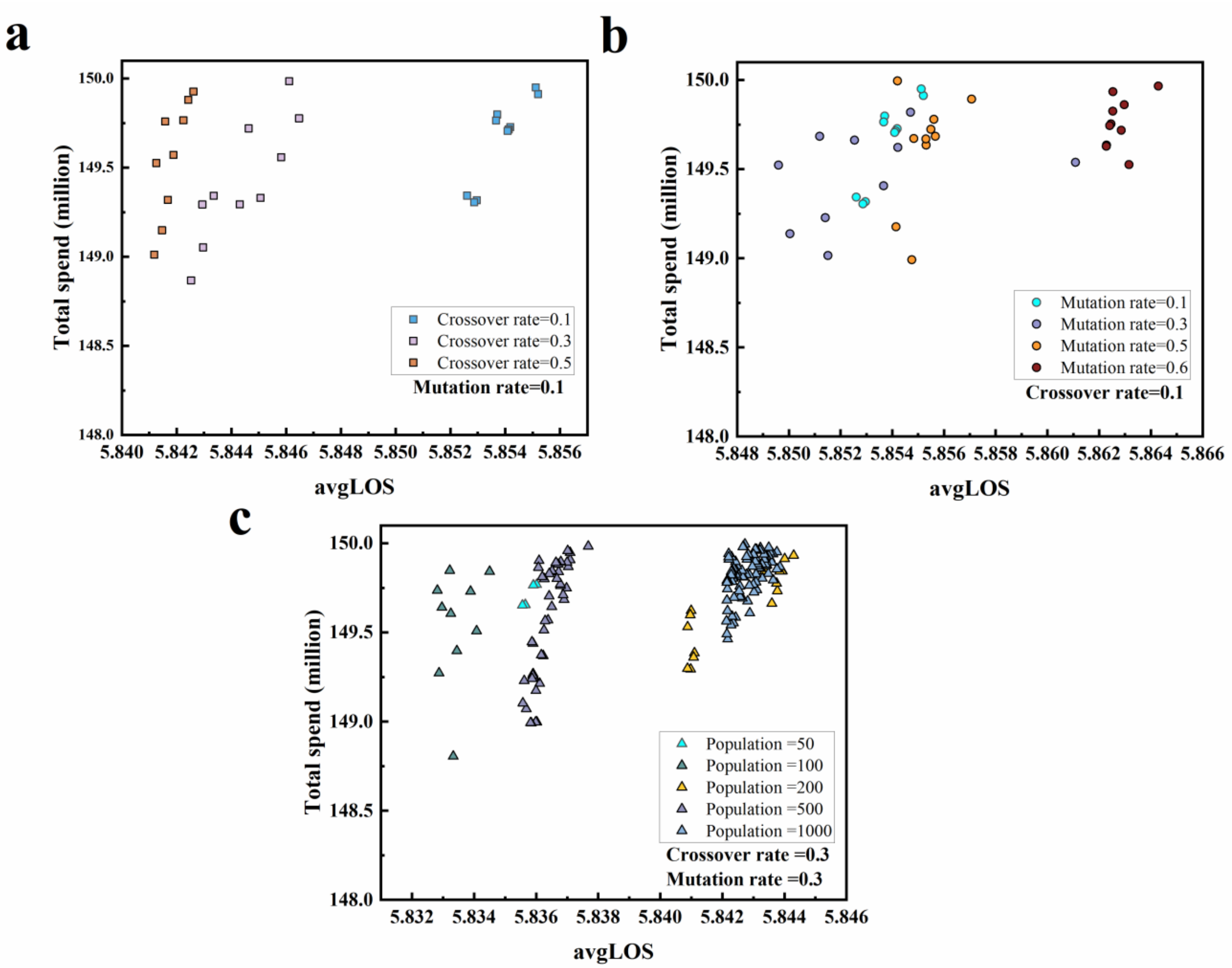

Fig. S10. Distribution and comparison of solution sets under different hyperparameter settings (a) crossover, (b) mutation, and (c) population in case 1.

Fig. S11.

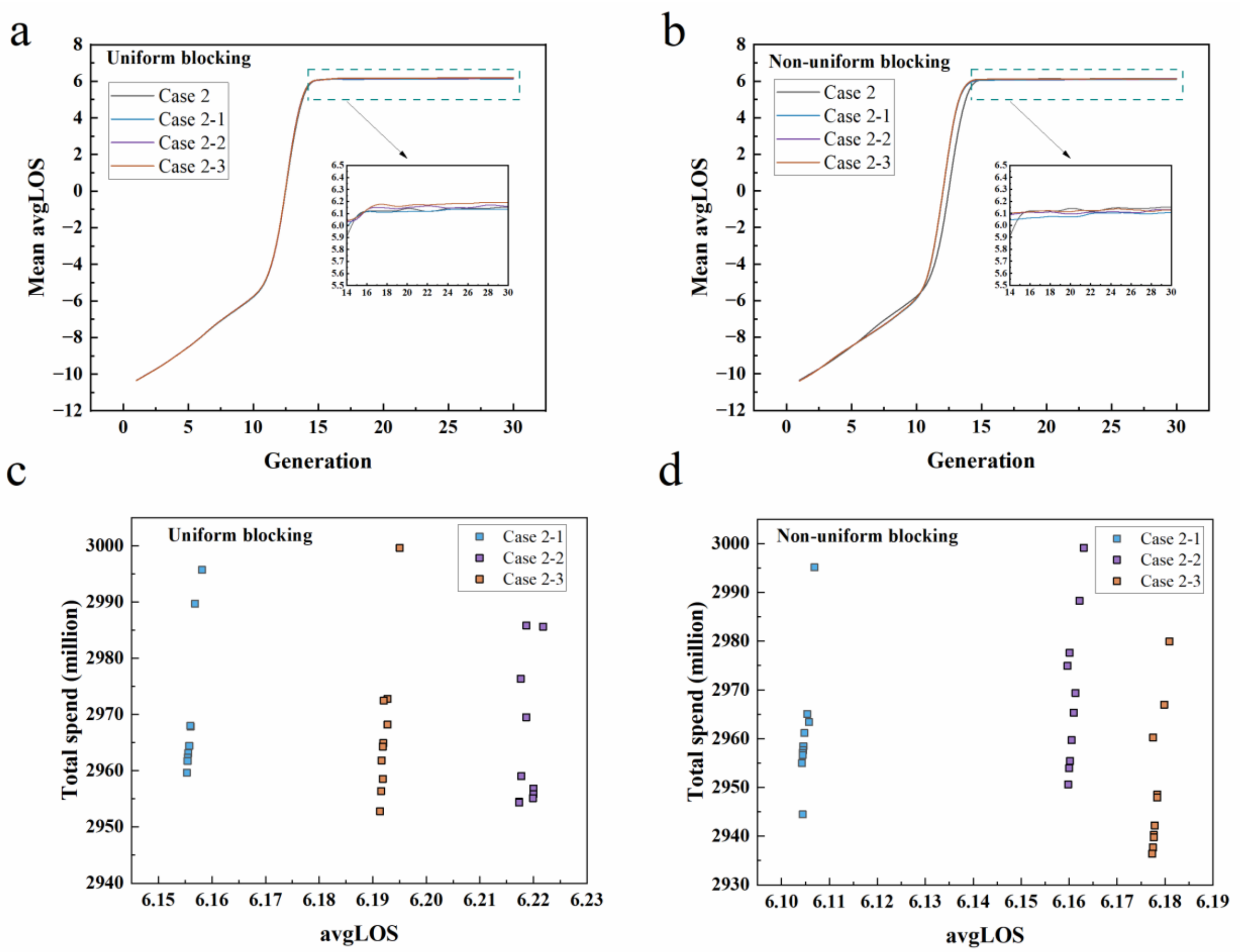

Fig. S11. Convergence and solution distribution of the NBGA framework under uniform and non-uniform blocking schemes. (a-b) Generational convergence trajectories of the mean avgLOS for three perturbed datasets (Cases 2-1, 2-2, and 2-3) under (a) uniform blocking and (b) dual-level non-uniform blocking. (c-d) Scatter plots of the final optimized solutions (total spend vs. avgLOS) across multiple runs, demonstrating the algorithm's stability and consistent high-quality output under (c) uniform and (d) non-uniform configurations.

Fig. S12.

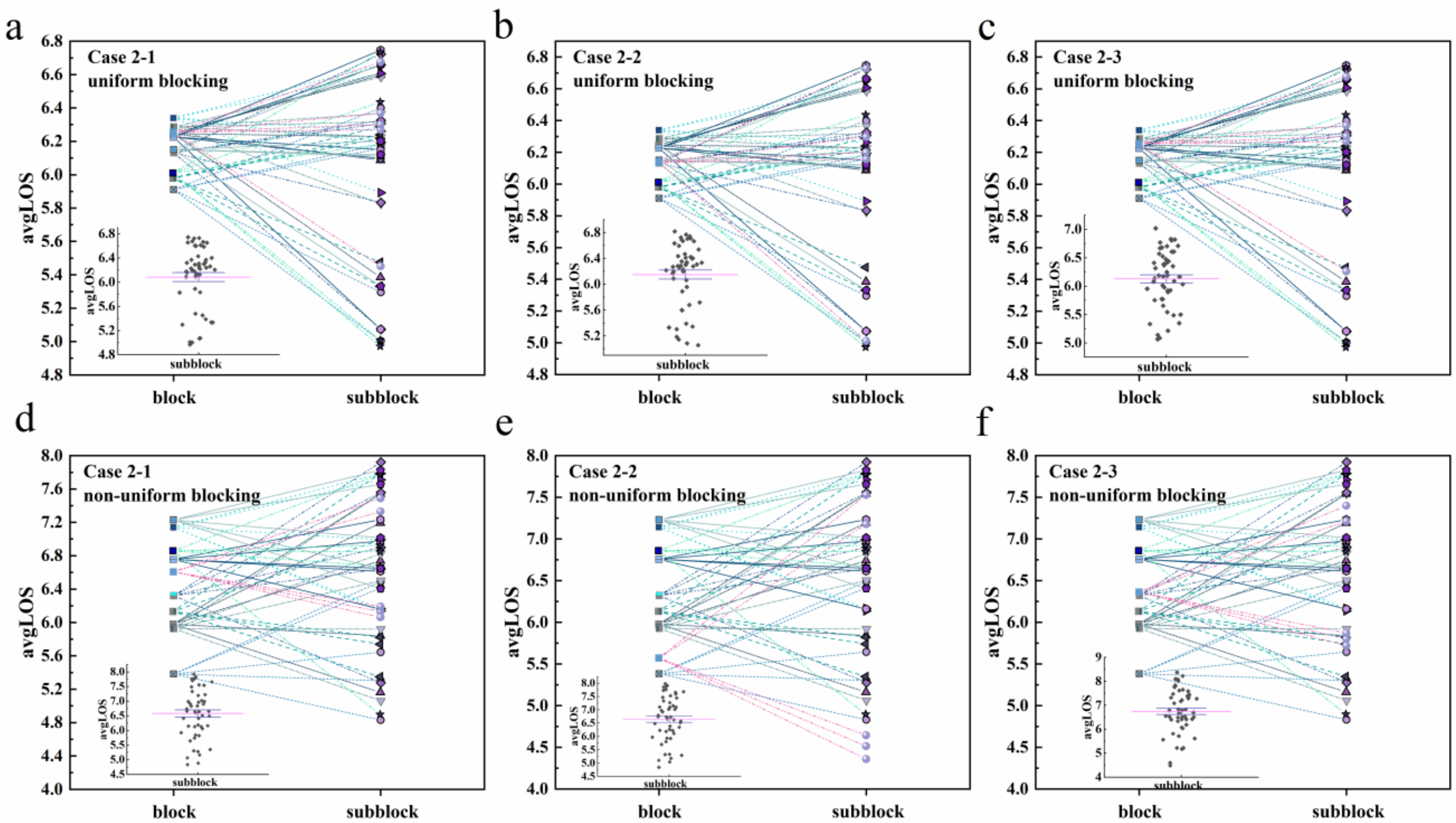

Fig. S12. Hierarchical condition (avgLOS) distribution across major blocks and subblocks. (a-c) Condition balancing from major blocks to minor subblocks under uniform partitioning for Cases 2-1, 2-2, and 2-3. (d-f) Condition balancing under dual-level non-uniform partitioning. Despite the severe structural asymmetry in subproblem sizes under the non-uniform scheme, the framework effectively regulates asset conditions, preventing extreme localized deterioration.

Fig. S13.

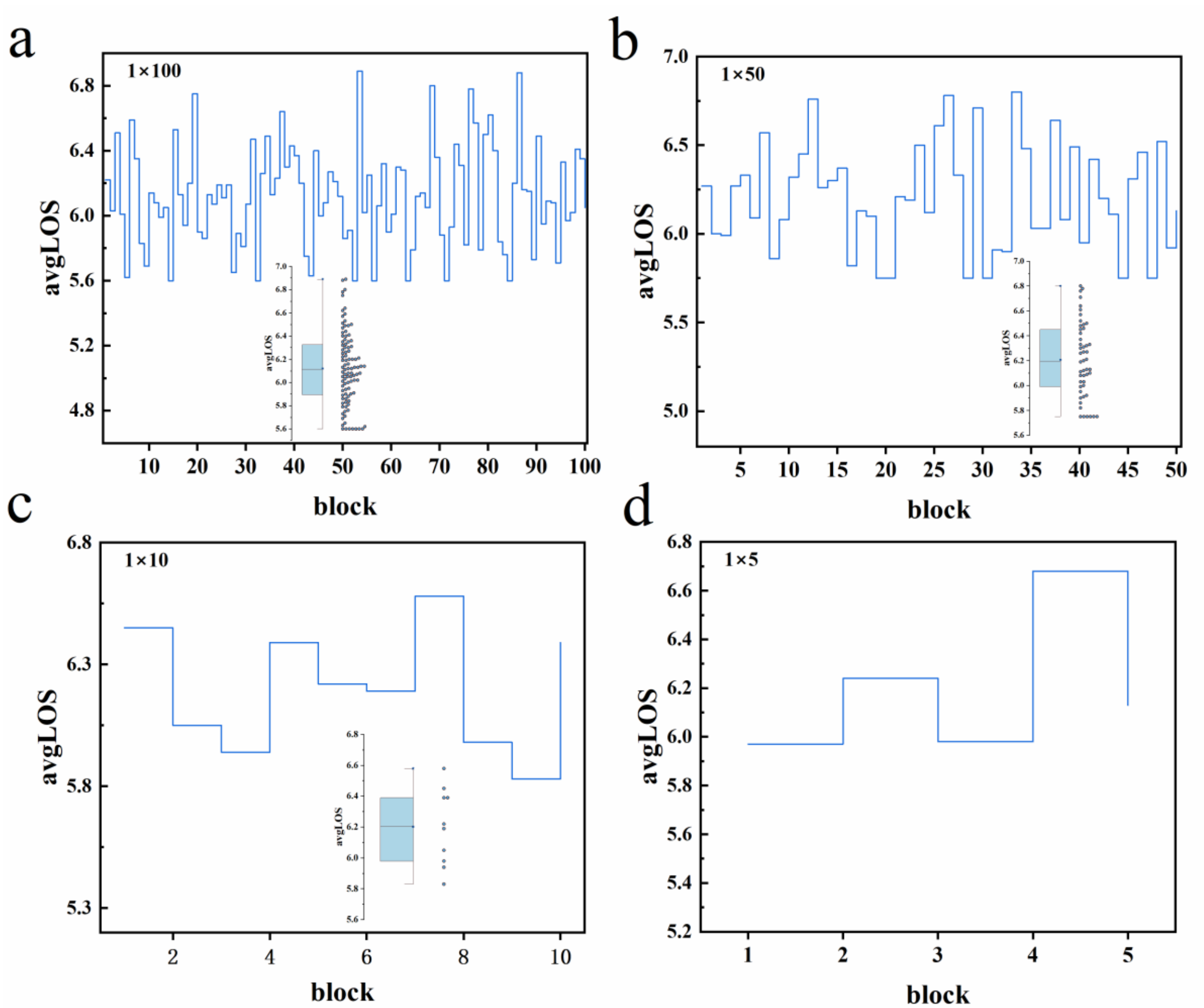

Fig. S13. Spatial distribution and statistical variation of avgLOS under different Single-Level nesting configurations. The four panels show the final avgLOS achieved in each block for different spatial granularities: (a) 100 blocks, (b) 50 blocks, (c) 10 blocks, and (d) 5 blocks. The line plots illustrate the block-to-block performance, while the inset boxplots summarize the overall statistical distribution (median, quartiles, and range) of avgLOS across all blocks for that configuration. The results show that while coarser granularities (e.g., 1×5) reduce high-frequency variations between adjacent blocks, all single-level configurations produce wide performance disparities, as evidenced by the large range in the boxplots. This supports the main text's finding (Fig. 2h) that single-level nesting can lead to significant spatial inequity.

Fig. S14.

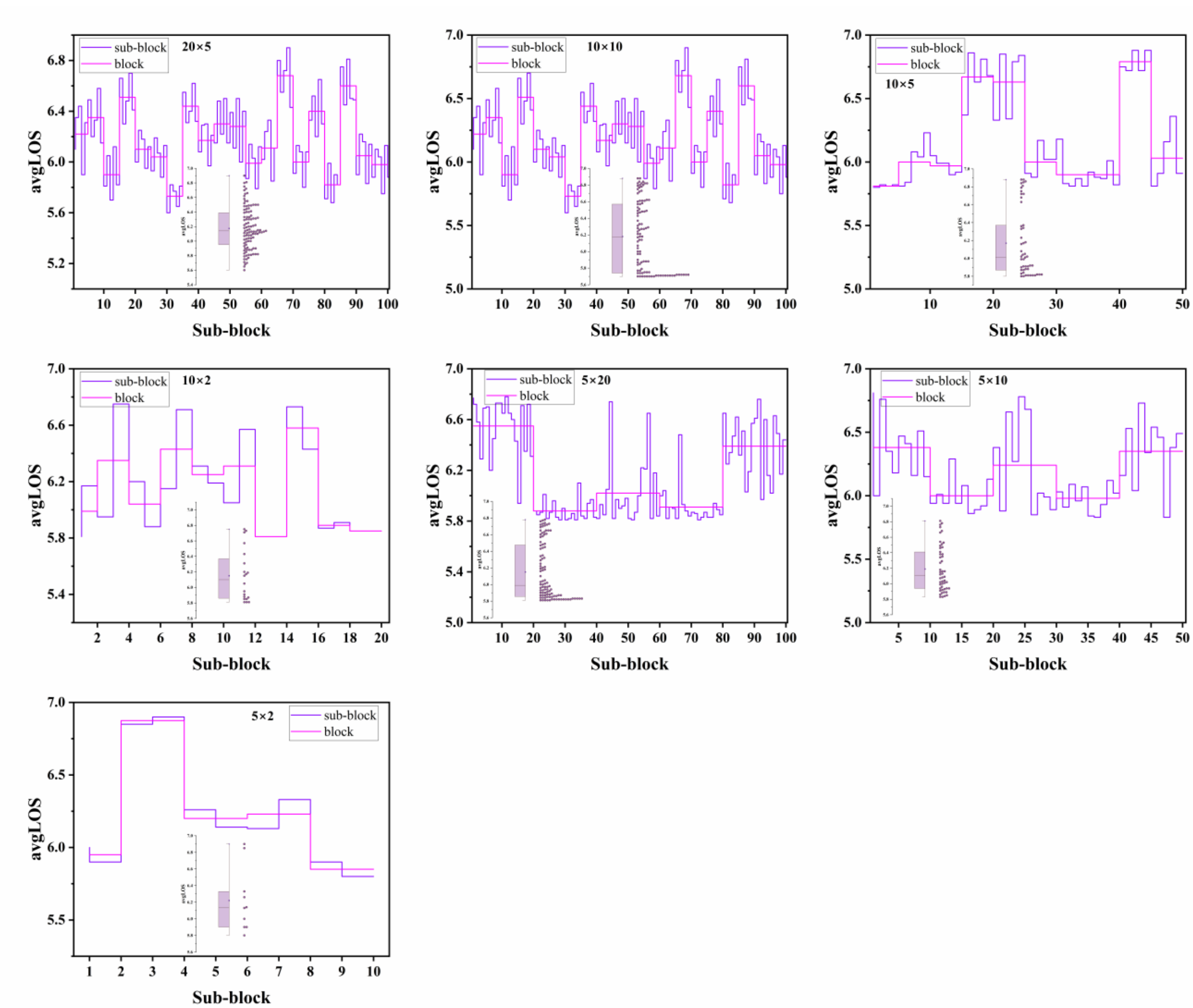

Fig. S14. Spatial distribution and statistical variation of avgLOS under different Double-Level nesting configurations. The seven panels show the final avgLOS for various two-level configurations (e.g., 20×5, 10×10, 10×5, 10×2, 5×20, 5×10, 5×2). The plots compare the performance of the parent-level 'block' (magenta line) with the performance of the 'sub-block' (purple line). The inset boxplots summarize the overall statistical distribution of avgLOS across all sub-blocks. These results demonstrate that sub-blocks within the same parent block tend to have similar performance levels, and the overall variation (shown in the boxplots) is generally tighter than in the single-level configurations. This supports the main text's finding (Fig. 2i) that two-level nesting provides a good balance between spatial granularity and performance consistency.

Fig. S15.

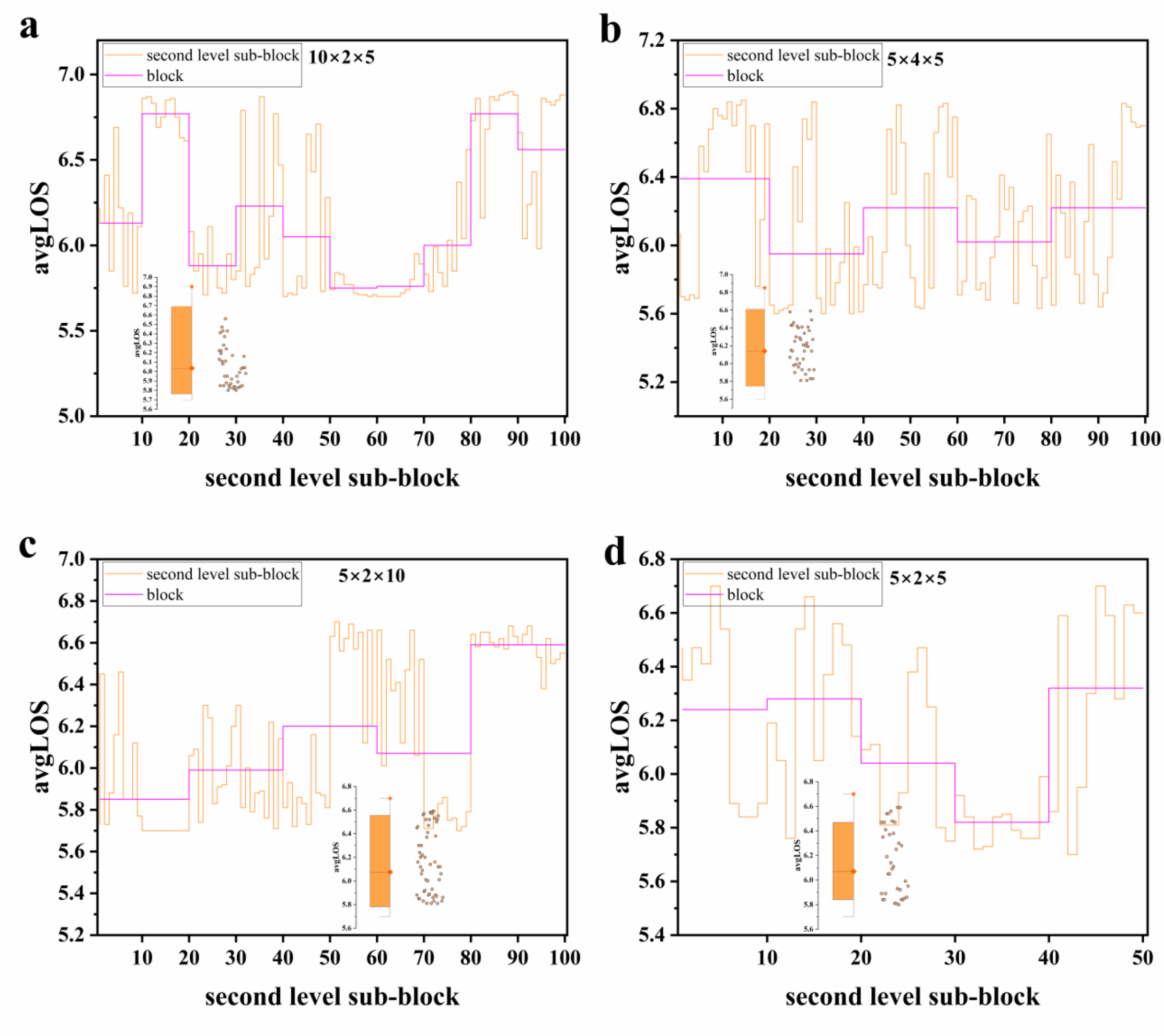

Fig. S15. Spatial distribution and statistical variation of avgLOS under different Triple-Level nesting configurations. The four panels show the final avgLOS for various three-level configurations: (a) 10×2×5, (b) 5×4×5, (c) 5×2×10, and (d) 5×2×5. The plots compare the performance of the highest-level parent 'block' (magenta line) with the performance of the lowest-level 'second level sub-block' (orange line). The inset boxplots summarize the overall statistical distribution of avgLOS across all lowest-tier sub-blocks. The results highlight that three-level nesting introduces finer segmentation, which increases performance variability among the lowest-tier sub-blocks, evidenced by the high-frequency fluctuations of the orange line and the wide ranges in the boxplots. This supports the main text's finding (Fig. 2j) that hyper-localization can lead to inconsistent service delivery and reduced overall efficiency.

Fig. S16.

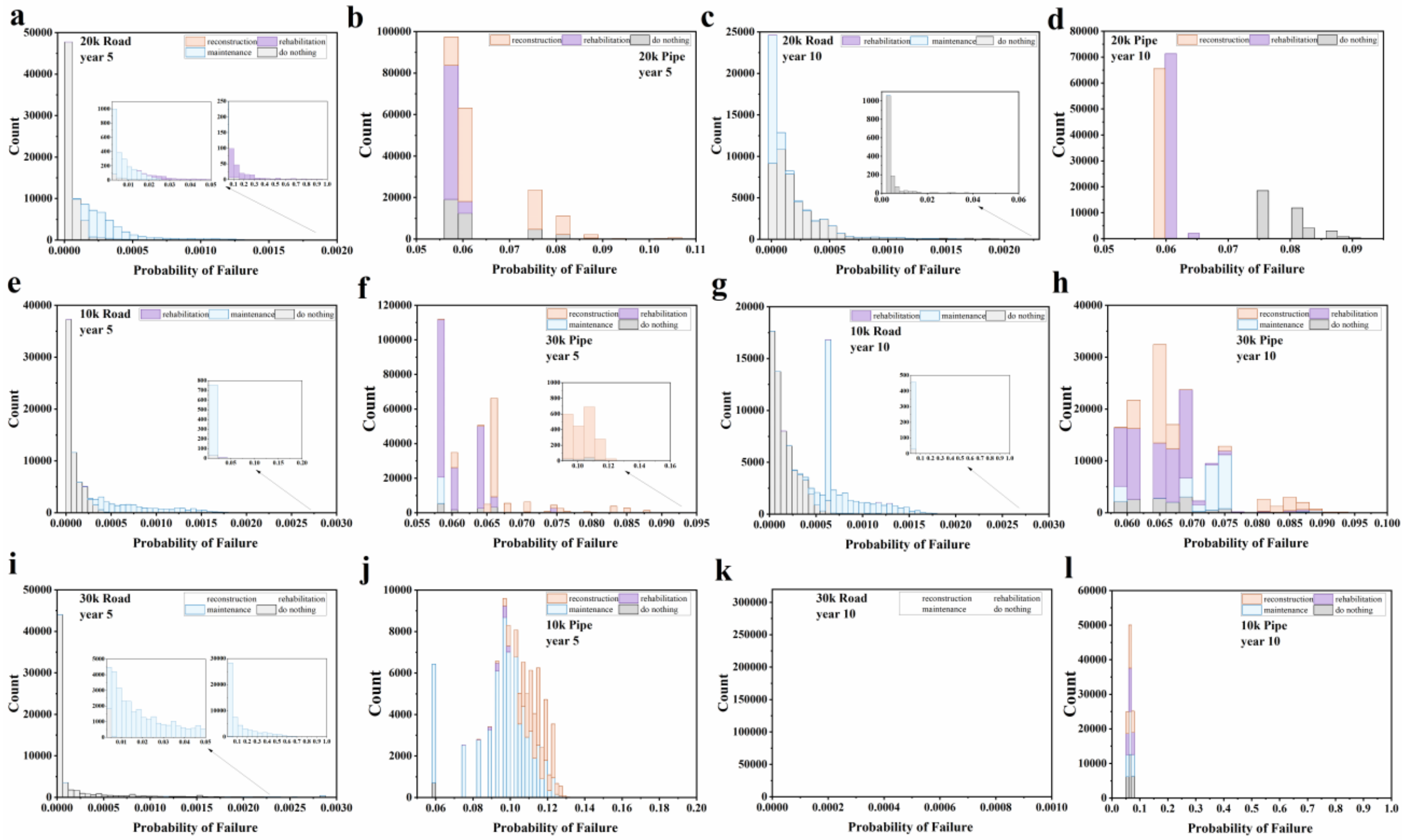

Fig. S16. Detailed Probability of Failure (PoF) distributions and maintenance decisions for all asset portfolios at Year 5 and Year 10. The twelve panels show the histograms of asset counts versus PoF for the three asset portfolios (rows): (a-d) 200k Road/200k Pipe, (e-h) 100k Road/300k Pipe, and (i-l) 30k Road/10k Pipe. For road assets (a, c, e, g, i, k), the distributions show a clear bifurcation. The main plots illustrate the vast majority of assets clustered at very low PoF (<0.03), while the insets reveal the 'long tail' of neglected assets that have degraded to very high PoF (>0.6). The stacked bars (legend in panel K) show the interventions applied. These plots quantify the 'over-maintenance' phenomenon, where low-risk road assets still receive 'maintenance' (blue) and 'rehabilitation' (purple) actions. Pipe assets (b, d, f, h, j, l) exhibit a similar risk profile, with the entire distribution clustered at a significantly higher PoF range. These data further substantiate the systemic inequity and asset bifurcation discussed in the main text (Fig. 4).

Fig. S17.

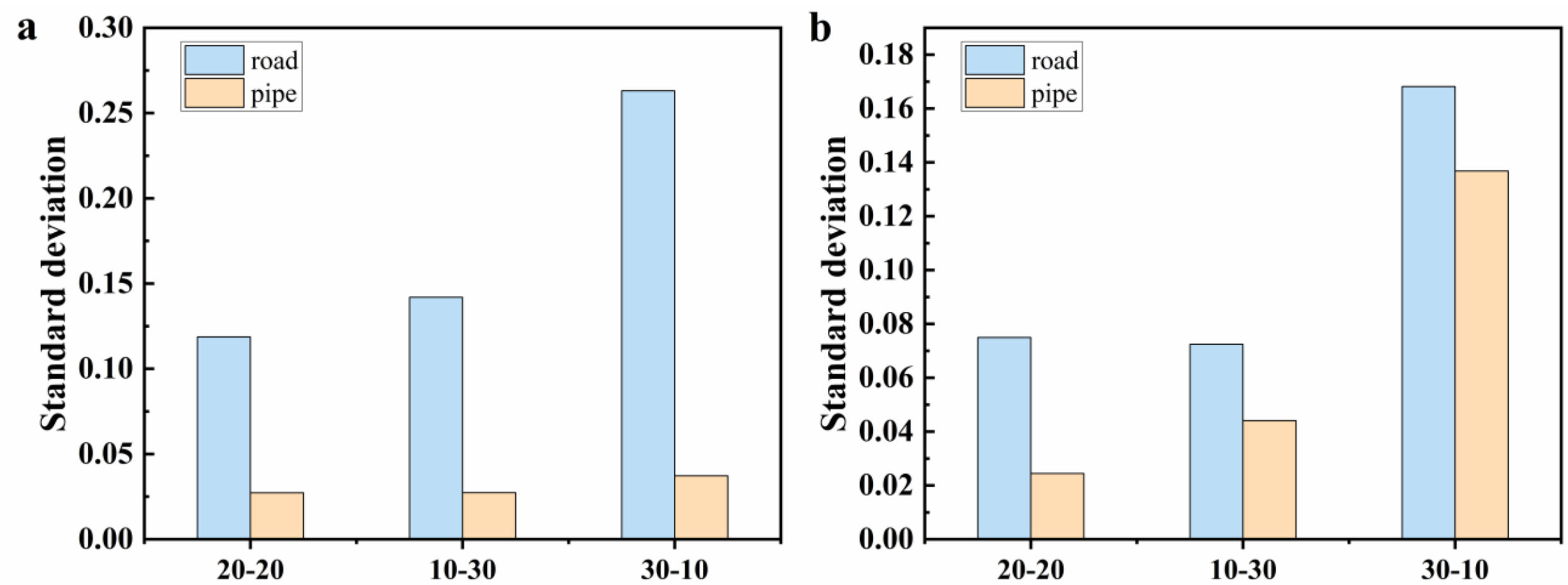

Fig. S17. Inter-annual volatility of equity metrics for the three asset portfolios. (a) Annual standard deviation of the Gini coefficient (Eq. S6) over the 10-year planning horizon. (b) Annual standard deviation of the Equity Coverage (Eq. S7) over the 10-year planning horizon. The x-axis represents the three asset portfolios (road/pipe compositions: 200k/200k, 100k/300k, and 300k/100k). Metrics are calculated separately for the road sub-network (blue) and the pipe sub-network (orange). The plots quantify the high instability (erratic fluctuations) of equity outcomes in the efficiency-focused model, providing the quantitative evidence referenced in the main text (Fig. 4d, 4f).

Fig. S18.

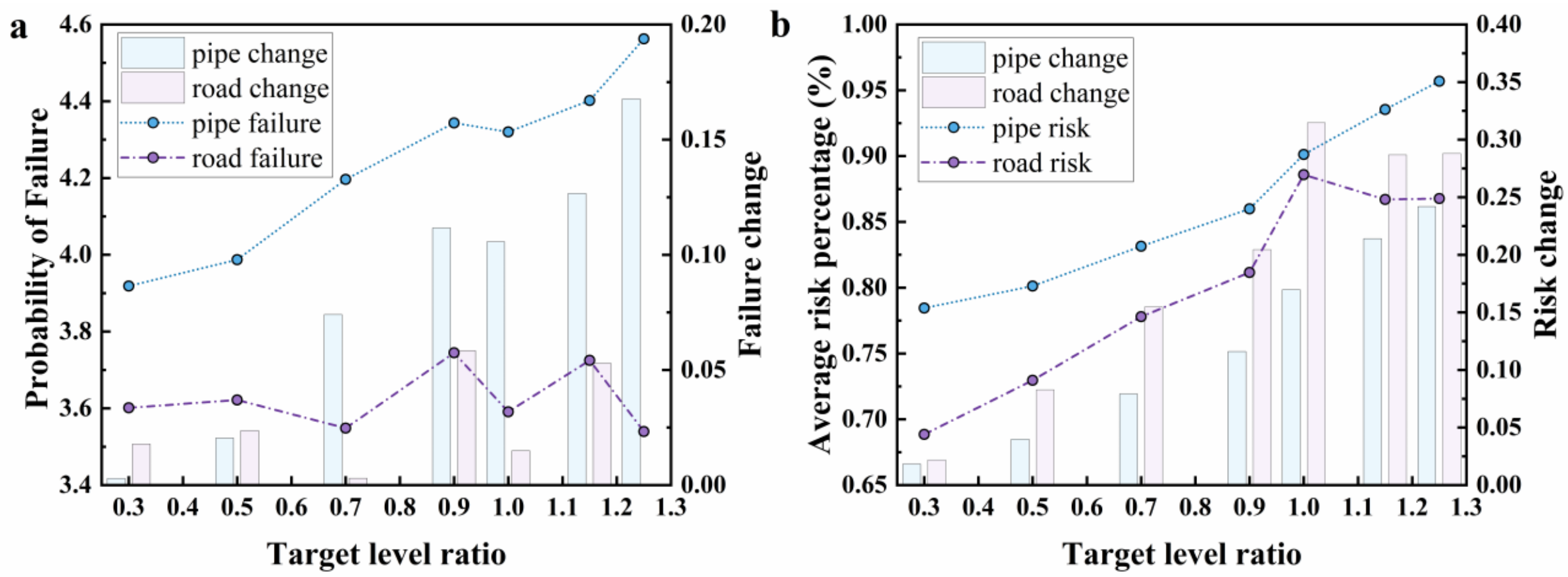

Fig. S18. Disaggregated impact of the Target Level Ratio (TLR) on road and pipe sub-network performance. (a) Average Probability of Failure (PoF) (lines, left axis) and PoF change (bars, right axis) for the road (purple/light purple) and pipe (blue/light blue) sub-networks as a function of the TLR. (b) Average Risk Percentage (lines, left axis) and risk change (bars, right axis) for the road and pipe sub-networks as a function of the TLR. These plots provide the detailed, asset-specific data behind the aggregated efficiency-equity trade-off analysis in the main text (Fig. 4g-i). They illustrate how enforcing higher equity baselines (increasing TLR) leads to a modest, quantifiable, and non-linear increase in both failure probability and average risk across both asset types.

Table S1.

Computational results of GA, Hybrid, NBGA and worst-first rolling-horizon plan (WF) for the three case studies

Case	Number of segments	Method	Best avgLOS	Execution time (second)	Crossover rate	Mutation rate	Coefficient of Variation
Case 1	13,610	GA	Unconvergence		0.3	0.3	N/A
		Hybrid GA	5.76	12,140	0.3	0.3	0.0010
		NBGA	5.83	6,300	0.3	0.3	0.0007
		WF	5.52	198	N/A	N/A	N/A
Case 2	100,000	GA	Unconvergence		0.3	0.3	N/A
		Hybrid GA	6.13	146,000	0.3	0.3	0.007
		NBGA	6.15	31,399	0.3	0.3	0.009
		WF	5.38	219	N/A	N/A	N/A
Case 3	1,000,000	GA	Unconvergence		0.3	0.3	N/A
		Hybrid GA	Out of memory		0.3	0.3	
		NBGA	6.19	291,400	0.3	0.3	
		WF	5.39	267	N/A	N/A	

Note: All computational experiments were executed on a workstation equipped with an Intel(R) Core(TM) i9-10900K CPU @ 3.70GHz. The population size was uniformly set to 100 for all algorithms across all case studies. The standard GA failed to yield valid results and is marked as "Unconvergence" because it could not reach steady-state convergence due to the complexity of the constraint landscape. The Hybrid GA is marked as "Out of memory" in Case 3 because the extreme scale of the million-asset decision space caused the population matrices to exceed the system's available RAM capacity, leading to computational termination.

Table S2.

The solution sets under different hyperparameter settings (crossover, mutation, and population) in case 1.

Case	Number of segments	Best avgLOS	Execution time (second)	Crossover rate	Mutation rate	Population
Case 1	13,610	5.85	2,220	0.1	0.1	100
		5.83	6,326		0.3	
		5.86	9,801		0.5	
		5.86	11,878		0.6	
		5.85	2,252	0.3	0.1	
		5.83	6,300		0.3	
		5.86	9,566		0.5	
		5.86	11,308		0.6	
		5.84	1,940	0.5	0.1	
		5.86	5,973		0.3	
		5.86	9,754		0.5	
		5.86	11,230		0.6	
		5.84	3,745	0.3	0.3	50
		5.84	7,101			200
		5.84	14,349			500
		5.84	34,244			1000

Note: All computational experiments were executed on a workstation equipped with an Intel(R) Core(TM) i9-10900K CPU @ 3.70GHz.

Table S3.

Summary of different nesting and blocking configurations for investigation (total number of assets in the network *n* = 100,000)

No.	Nesting	Block		Sub-block		Second level sub-block		Time (second)	avg LOS
		n_b	$\rho_{pb}(n_{pb})$	n_b	$\rho_{ob1}(n_{ob1})$	n_b	$\rho_{ob2}(n_{ob2})$		
1	Single	100	1% (1,000)	N/A	N/A	N/A	N/A	3,4384	6.08
2		50	2% (2,000)	N/A	N/A	N/A	N/A	3,4232	6.21
3		20	5% (5,000)	N/A	N/A	N/A	N/A	3,8241	6.23
4		10	10% (10,000)	N/A	N/A	N/A	N/A	4,0236	6.20
5		5	20% (20,000)	N/A	N/A	N/A	N/A	4,4197	6.19
6	Double	20	5% (5,000)	5	1% (1,000)	N/A	N/A	3,7235	6.17
7		10	10% (10,000)	10	1% (1,000)	N/A	N/A	3,6814	6.18
8		10	10% (10,000)	5	2% (2,000)	N/A	N/A	3,2851	6.17
9		10	10% (10,000)	2	5% (5,000)	N/A	N/A	3,6619	6.15
10		5	20% (20,000)	20	1% (1,000)	N/A	N/A	4,0629	6.15
11		5	20% (20,000)	10	2% (2,000)	N/A	N/A	3,6475	6.19
12		5	20% (20,000)	4	5% (5,000)	N/A	N/A	3,1399	6.24
13		5	20% (20,000)	2	10% (10,000)	N/A	N/A	3,5806	6.22
14	Triple	10	10% (10,000)	2	5% (5,000)	5	1% (1,000)	4,1611	6.19
15		5	20% (20,000)	4	5% (5,000)	5	1% (1,000)	3,5915	6.16
16		5	20% (20,000)	2	10% (10,000)	10	1% (1,000)	3,8921	6.14
17		5	20% (20,000)	2	10% (10,000)	5	2% (2,000)	4,0196	6.14
18		5	20% (20,000)	2	10% (10,000)	2	5% (5,000)	4,3827	6.18

Note: n_b = number of total blocks or subblocks; $\rho_{pb} =$ proportion of assets in a parent block; n_{pb} = number of assets in a parent block; $\rho_{ob} =$ proportion of assets in a subblock (offspring block); and n_{ob} = number of assets in a subblock (offspring block).
